# Knock down of transforming growth factor beta improves expressions of co-stimulatory molecules, type I interferon-regulated genes, and pro-inflammatory cytokine in PRRSV-inoculated monocyte-derived macrophages

**DOI:** 10.1186/s12917-023-03760-8

**Published:** 2024-08-03

**Authors:** Dante Fabros, Wasin Charerntantanakul

**Affiliations:** Research Laboratory for Immunity Enhancement in Humans and Domestic Animals, Program of Biotechnology, Faculty of Science, Maejo University, Chiang Mai, 50290 Chiang Mai, Thailand

**Keywords:** Porcine reproductive and respiratory syndrome virus, Transforming growth factor beta, Antisense, Gene knockdown, Innate immunity

## Abstract

Porcine reproductive and respiratory syndrome virus (PRRSV) induces a poor innate immune response following infection. This study evaluates the effects of transforming growth factor beta 1 (TGFβ1) up-regulated by PRRSV on gene expressions of co-stimulatory molecules, type I interferon (IFN), type I IFN-regulated genes (IRGs), pattern recognition receptors, and pro-inflammatory cytokines in PRRSV-inoculated monocyte-derived macrophages (MDMs). Phosphorothioate-modified antisense oligodeoxynucleotides (AS ODNs) specific to various regions of porcine TGFβ1 mRNA were synthesized, and those specific to the AUG region efficiently knockdown TGFβ1 mRNA expression and protein translation. Transfection of TGFβAS ODNs in MDMs inoculated with either classical PRRSV-2 (cPRRSV-2) or highly pathogenic PRRSV-2 (HP-PRRSV-2) significantly reduced TGFβ1 mRNA expression and significantly increased mRNA expressions of CD80, CD86, IFNβ, IRGs (i.e. IFN regulatory factor 3 (IRF3), IRF7, myxovirus resistance 1, osteopontin, and stimulator of IFN genes), Toll-like receptor 3, and tumor necrosis factor-alpha. Transfection of TGFβAS ODNs in MDMs inoculated with HP-PRRSV-2 also significantly increased mRNA expressions of IFNα, IFNγ, and 2’-5’-oligoadenylate synthetase 1. The quantity of PRRSV-2 RNA copy numbers was significantly reduced in MDMs transfected with TGFβAS ODNs as compared to untransfected MDMs. Recombinant porcine TGFβ1 (rTGFβ1) and recombinant porcine IFNα (rIFNα) sustained and reduced the yields of PRRSV-2 RNA copy numbers in PRRSV-2 inoculated MDMs, respectively. These findings demonstrate a strategy of PRRSV for innate immune suppression via an induction of TGFβ expression. These findings also suggest TGFβ as a potential parameter that future PRRSV vaccine and vaccine adjuvant candidates should take into consideration.

**Supplementary Information:**

The online version contains supplementary material available at 10.1186/s12917-023-03760-8.

## Introduction

Porcine reproductive and respiratory syndrome virus (PRRSV) causes global economic loss of swine industry. PRRSV is an enveloped RNA virus under family *Arteriviridae*, order *Nidovirales*. Its genome is approximately 15 kb in size, consisting of 11 open reading frames (ORFs). The virus is classified as PRRSV-1 (formerly European genotype) and PRRSV-2 (formerly North American genotype). Both PRRSV species share up to 60% nucleotide sequence homology and comprise classical PRRSV (cPRRSV) strains and highly pathogenic PRRSV (HP-PRRSV) strains [1].

Porcine myeloid antigen (Ag)-presenting cells (APCs), e.g., monocytes [2], macrophages [3] and monocyte-derived dendritic cells [4] are the primary target for PRRSV infection. PRRSV contains several proteins, i.e., nonstructural protein 1 (Nsp1) [5], Nsp2 [6], Nsp4 [7], Nsp5 [8], Nsp11 [9], glycoprotein 5 (GP5) [10], and nucleocapsid (N) protein [11] that mediate down-regulation of type I interferon (IFN)-regulated gene (IRG) expression in infected APCs. These proteins, together with yet unidentified PRRSV proteins, involve in PRRSV suppression of signaling molecule and transcription factor activation, e.g. retinoic-acid induced gene-1 (RIG-1) [9], mitochondrial antiviral signaling protein [9], IFN regulatory factor 3 (IRF3) [5], STAT1 [8], extracellular signal-regulated kinase (ERK) [12], and NFκB [13]; PRRSV-mediated degradation of CREB-binding protein [14]; and PRRSV inhibition of nuclear translocation of STAT1 and STAT2 [8]. In contrast to the down-regulation of IRG expression, PRRSV up-regulates interleukin-10 (IL-10) expression [2, 15]. Weak IRG and robust IL-10 expressions contribute to a weak induction of pro-inflammatory innate immune defense, e.g., phagocytic and antiviral activities, Ag presentation, and pro-inflammatory cytokine and immune-related molecule expressions of infected myeloid APCs; weak and delayed induction of adaptive cytotoxic T cell and T helper 1 (Th1) cell responses; and promotion of regulatory T cell (Treg) differentiation [4, 16, 17]. These poor pro-inflammatory innate and adaptive cell-mediated immune (CMI) responses facilitate PRRSV survival and clinical manifestations.

Apart from the up-regulation of IL-10, PRRSV up-regulates transforming growth factor beta (TGFβ) expression in infected myeloid APCs [18], co-cultivated peripheral blood mononuclear cells (PBMCs) [19], and lymphoid tissues and lungs of PRRSV-infected pigs [20]. The role of PRRSV-induced TGFβ overexpression on immune protection against PRRSV has never been studied to date. TGFβ has been reported to elevate the viability of PRRSV-infected cells, which contribute to increasing PRRSV survival [21]. The cytokine reportedly down-regulates CD14, MHCII, IL-6, and tumor necrosis factor alpha (TNFα) expressions in porcine monocyte-derived macrophages (MDMs) [22]. Studies in mice have shown that TGFβ down-regulates CD14 expression in lipopolysaccharide (LPS)-stimulated macrophages, resulting in suppression of MyD88-dependent signaling pathway [23, 24]. The cytokine also suppresses IL-12p40, and CD40 expressions in macrophages [24]; Th1 cell differentiation, Th1-mediated inflammatory response and expression of IFNγ, IL-2, and IL-4 [25]; and activation of macrophage, dendritic cells (DCs), and natural killer cells [24]. On the other hand, TGFβ promotes Treg differentiation through up-regulation of Foxp3 and Smad3 expression [25]. In mammals, there exist three isoforms of TGFβ. Among them, TGFβ1 is the most abundant isoform and is responsible for a great variety of specific responses to TGFβ [26].

In this study, we aim to investigate the effects of PRRSV-induced TGFβ overexpression on immune-related gene responses in PRRSV-inoculated MDMs. We employed phosphorothioate-modified antisense (AS) oligodeoxynucleotides (ODNs) specific for porcine TGFβ1 mRNA to knock down its expression. Our findings report that TGFβ plays roles in down-regulating gene expressions of co-stimulatory molecules, type I IFN, IRGs, and pro-inflammatory cytokines in PRRSV-inoculated MDMs. Our findings suggest potential strategies to improve innate and adaptive CMI responses to future PRRSV vaccines and vaccine adjuvants.

## Materials and methods

### PRRSV

Thai cPRRSV-2 (01NP1) [27] and HP-PRRSV-2 (10PL1) [28] were propagated in MARC-145 cells grown in MEM^++^ comprising MEM (Caisson Laboratories, Smithfield, UT), 10% heat-inactivated fetal bovine serum (FBS; Capricorn Scientific GmbH, Germany), penicillin (100 IU/ml), streptomycin (100 µg/ml), and amphotericin B (250 ng/ml) (all from Gibco, NY) to 10^6^ TCID_50_/ml. Supernatants from uninoculated MARC-145 cell lysate were used as mock Ag.

### Animals

Eight 24-week-old PRRSV-seronegative crossbred pigs (Large White/Landrace x Duroc) were the sources of PBMCs. They were housed at the swine research farm, faculty of animal science and technology, Maejo University.

### Phosphorothioate-modified ODNs

All phosphorothioate-modified ODNs were synthesized by Integrated DNA Technologies (IDT, Singapore). Their sequences are detailed in Table 1.

**Table 1:** Sequences of antisense (AS), sense (S), and scramble (Scr) phosphorothioate-modified ODNs used in this study

ODNs	Sequence (5’-3’)	Complementary to position on TGFβ1 mRNA (NM_214015.2)	Target region
TGFβAS1	AGCCCCGAAGGCGGCATG	-2-16	AUG region
TGFβAS2	GTTGTACAGAGCCAGGAC	1015–1032	Coding region
TGFβAS3	GCCATGAGGAGCAGGAAG	768–785	Coding region
TGFβAS4	CACCAGCTCCATGTCGAT	106–123	Coding region
TGFβS1	CATGCCGCCTTCGGGGCT		Negative control
TGFβS2	GTCCTGGCTCTGTACAAC		Negative control
TGFβS3	CTTCCTGCTCCTCATGGC		Negative control
TGFβS4	ATCGACATGGAGCTGGTG		Negative control
Scr1	GCCGCTTGCTCGCGCCTA		Scramble control
Scr2	CGTCGAATCGCCGGAGTA		Scramble control
Scr3	CGTCGAATCGCCGGAGTA		Scramble control
Scr4	CCGTACTACATTCGTCAC		Scramble control

### Optimization of real-time PCR conditions

Isolation of PBMCs was conducted as described previously [29]. Briefly, PBMCs were isolated from whole blood by density gradient centrifugation using Lymphoprep™ (Stemcell Technologies, Norway). Contaminating erythrocytes were lysed by cold lysis buffer (1 mM EDTA, 0.156 M ammonium chloride and 10 mM sodium bicarbonate). PBMCs were resuspended in RPMI^++^ (RPMI-1640 with L-glutamine (Caisson Laboratories), 10% heat-inactivated FBS, penicillin (100 IU/ml), streptomycin (100 µg/ml) and amphotericin B (250 ng/ml)). PBMC suspension (200 µl; 2 × 10^6^ cells) was seeded onto 96-well flat-bottom plates (Nunc, Denmark), and received 50 µL of inducers (a mixture of polyinosinic:polycytidylic acid (poly I:C; 10 µg/ml final conc.) and LPS from *E. coli* O111:B4 (5 µg/ml final conc; both from Sigma, St. Louis, MO)). The final concentrations of poly I:C and LPS used in this study were the least concentrations that could induce detectable mRNA expressions of all immune-related genes of interest. Cells were stimulated for 18 h (37 °C, humidified 5% CO_2_) prior to RNA isolation.

Total RNA was isolated using a NucleoSpin® RNA kit (Macherey-Nagel, Germany). The quality and quantity of RNA were evaluated by a Nanodrop 2000/2000c spectrophotometer (NanoDrop Technologies, Montchamin, DE). All RNA samples had A260/230 and A260/280 between 1.8 and 2.2 and 2.0-2.2, respectively. The integrity of RNA was determined by denaturing agarose gel electrophoresis followed by ethidium bromide staining. Reverse transcription was carried out, using RevertAid™ First Strand cDNA synthesis kit (Thermoscientific, Lithuania). The reaction used 1,000 ng of pooled total RNA as template, and a mixture of oligo-dT and random hexamers as primers. cDNA was stored at -20 °C until real-time polymerase chain reaction (PCR).

Real-time PCR was performed on Applied Biosystems 7500 Fast thermal cycler. A total reaction volume of 20 µl comprised 2 µl serial 5-fold dilutions of pooled cDNA template (starting at 1 µg), 10 µl SYBR® Green real-time PCR master mix (Toyobo, Japan), and varying concentrations (200–500 nM) of primer pairs for CD80, CD86, IFNα, IFNβ, IFNγ, IL-1β, IL-6, IL-10, IRF3, IRF7, myxovirus resistance 1 (Mx1), 2’-5’-oligoadenylate synthetase 1 (OAS1), osteopontin (OPN), stimulator of interferon genes (STING), Toll-like receptor 3 (TLR3), TLR4, TLR7, TLR8, TLR9, TGFβ1 and TNFα (Supplementary Table 1). All reactions were set up in duplicate. The PCR condition was 95 °C (10 min); and 40 cycles of 94 °C (15s), designated annealing temperature at 50–60 °C (30s), and 72 °C (30s), followed by melting curve analysis and agarose gel electrophoresis of PCR products. Band intensities were documented under ultraviolet light (GelMax™ Imager, UVP, CA). A nuclease-free water was included as no template control in every run.

### Preparation of MDMs

**Fig. 1:**
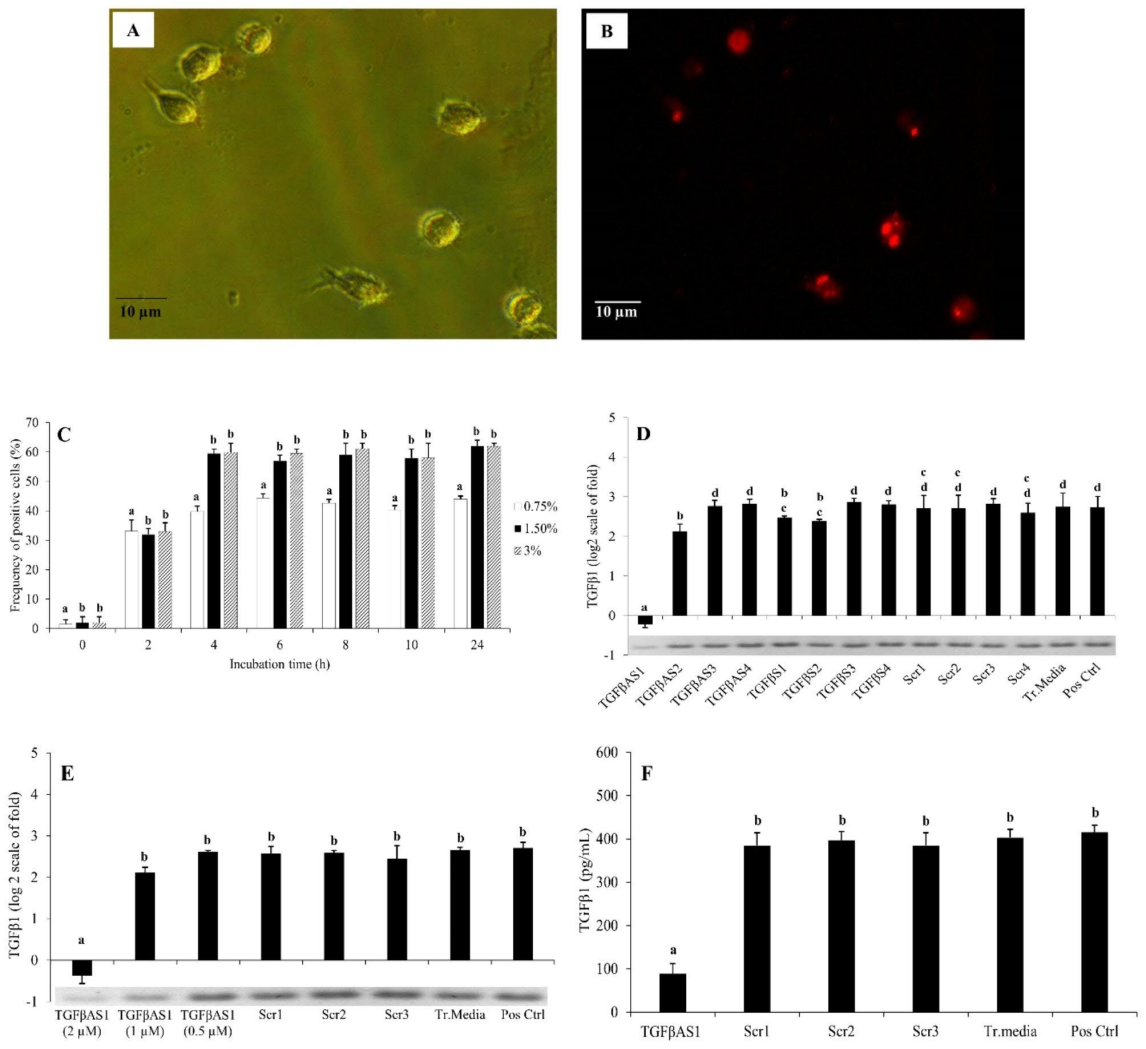
Optimization and validation of knockdown of porcine TGFβ1 mRNA expression and protein translation by AS ODNs. **A**) MDMs under bright-field microscopy. **B**) MDMs uptake of fluorescent-labeled siRNA under immunofluorescent microscopy. **C**) MDMs uptake of fluorescent-labeled siRNA complexed with different concentrations of transfection reagent and transfection period. **D**) Effect of TGFβ1 antisense (AS1-4), sense (S1-4) and scramble (Scr1-4) phosphorothioate-modified ODNs on the expression of TGFβ1 mRNA in MDMs stimulated with a mixture of poly I:C and LPS. Band intensities (Additional file 1) indicate the quality of TGFβ1 knockdown. **E**) Optimization of TGFβAS1 concentration for TGFβ1 mRNA knockdown on MDMs transfected with TGFβAS1 (0.5, 1, or 2 µM) and stimulated with a mixture of poly I:C and LPS. Band intensities (Additional file 2) indicate the quality of TGFβ1 knockdown. **F**) TGFβ1 protein levels in MDMs transfected with TGFβAS1 (2 µM) and stimulated with a mixture of poly I:C and LPS. In all figures, error bars indicate the standard deviation (SD). Mean differences of TGFβ1 gene expression or protein translation among groups were tested by one-way ANOVA, followed by Tukey HSD test. Mean differences of percentages of fluoresced cells among groups at time points were tested by one-way repeated measures ANOVA, followed by Tukey HSD test. Different letters indicate significant differences. P < 0.05 was set as a statistically significant level

The preparation of MDMs was conducted as described previously [30]. Briefly, PBMC suspension (100 µl; 10^6^ cells) was seeded onto a 96-well flat bottom plate and incubated for 4 h (37 °C, humidified 5% CO_2_). Non-adherent cells were removed, and adherent monocytes were washed twice with 150 µl pre-warmed (37 °C) RPMI^++^. The cultures were incubated for 7 days for MDM differentiation (Fig. 1A). Fresh RPMI^++^ (150 µl) was replaced every other day. On day 7, RPMI^++^ was totally removed and replaced with reduced serum media (Opti-MEM^®^ I, Gibco).

### Transfection of MDMs with AS-ODNs

Transfection was carried out following the guideline of Lipofectamine^™^ RNAiMax (Invitrogen, Carlsbad, CA) with recommended small interfering RNA (siRNA, BLOCK-iT™ Alexa Fluor^®^ Red Fluorescent control, Invitrogen). In brief, different mixtures of Lipofectamine^™^ RNAiMax in Opti-MEM^®^ I (v/v) and 2 µM siRNA suspended in Opti-MEM^®^ I were added to the wells containing MDMs. Cell uptake of fluorescent-labeled siRNA was observed under the immunofluorescent microscope (Nikon Eclipse Ti, Japan). Frequencies of immunofluorescent-positive cells were identified using automatic measurement for cell counting (NIS-elements software ver. 3.22, Nikon, Japan). Cell viability was determined by trypan blue staining in parallel. Optimal concentration of transfection reagent and optimal transfection period were determined.

### Evaluation of TGFβAS1-4 efficiency on TGFβ1 mRNA knockdown

TGFβAS mixtures containing 2 µM of either TGFβAS1, TGFβAS2, TGFβAS3, or TGFβAS4 in transfection media (1.5% v/v of Lipofectamine™ RNAiMAX in Opti-MEM® I) were incubated at RT for 30 min. Then, 20 µl of the mixtures were added to wells containing MDMs in 100 µl of Opti-MEM® I. The cultures were mixed gently by rocking the plates back and forth for 5 min and incubated further for 4 h (37 ^o^C, humidified 5% CO_2_). The media were then removed and replaced with 200 µl of RPMI^++^ and 50 µl of inducers. Plates were incubated for 12 h (37 ^o^C, humidified 5% CO_2_), then the cells were harvested, washed with PBS, and evaluated for TGFβ1 mRNA expression by real-time PCR. Untransfected MDMs stimulated or not with inducers served as positive and negative controls, respectively. MDMs treated with scramble (Scr) ODNs or transfection media prior to stimulation with inducers served as Scr and transfection media controls, respectively.

For the determination of mRNA expression levels of TGFβ1 and other immune-related genes, 200 ng of total RNA was used as the template for cDNA synthesis. The threshold cycles (C_T_) of all genes were used for the calculation of gene expression by 2^(-ΔΔC_T_) method. The expressions of TGFβ1 and other immune-related genes were normalized to the geometric average of RPL32 (ribosomal protein L32) and YWHAZ (tyrosine 3-monooxygenase/tryptophan 5-monooxygenase activation protein, zeta) and calibrated to that in the negative control. High expression stability of RPL32 and YWHAZ in porcine MDMs stimulated or not with inducers has been reported [31]. The expression levels of all immune-related genes were transformed into log 2 scale.

### Evaluation of TGFβAS1 specificity

TGFβAS1 (2 µM) transfection media was prepared and transfected to MDMs as described above. Cells were stimulated with 50 µl of inducers. Cell culture supernatants were collected for subsequent determination of TGFβ1 protein levels by enzyme-linked immunosorbent assay (ELISA) (Porcine TGF Beta 1 PicoKine™ ELISA kit, Boster Biological Technology, Pleasanton, CA). Inadvertent knock down of immune-related genes, i.e. CD80, CD86, IFNα, IFNβ, IFNγ, IL-1β, IL-6, IL-10, IRF3, IRF7, Mx1, OAS1, OPN, STING, TLR3, TLR4, TLR7, TLR8, TLR9, and TNFα was determined by real-time PCR. Positive, negative, Scr, and transfection media controls were included.

### Evaluation of TGFβ1 knockdown effects on immune-related gene expressions in MDMs inoculated with cPRRSV-2 and HP-PRRSV-2

TGFβAS1 (2 µM) in transfection media were transfected to MDMs as described above. Subsequently, transfection media were removed and replaced with 100 µl RPMI^++^ and 100 µl of either cPRRSV-2 or HP-PRRSV-2 (equivalent to multiplicity of infection (m.o.i) of 1). The cultures were incubated for 48 h (37 °C, humidified 5% CO_2_), then received 50 µl of inducers. The cultures were incubated further for 12 h (37 °C, humidified 5% CO_2_) prior to RNA isolation. Cell culture supernatants were collected for the determination of TGFβ1 protein levels by ELISA. Expressions of immune-related genes were determined by real-time PCR. Controls included MDMs receiving mock Ag plus inducers (mock control); MDMs receiving PRRSV-2 and inducers (PRRSV-2-inoculated control); and MDMs treated with transfection media alone (without TGFβAS1), inoculated with PRRSV-2, and stimulated with inducers (PRRSV-2-inoculated/transfection media control). Untreated MDMs receiving culture media in the presence or absence of inducers served as positive and negative controls, respectively. Cell viability was determined at the end of the transfection period, PRRSV-2 inoculation, and inducer stimulation using trypan blue.

### Evaluation of TGFβ1 knockdown effects on PRRSV RNA yields in MDMs inoculated with cPRRSV-2 and HP-PRRSV-2

MDMs were transfected with TGFβAS1 (2 µM) in transfection media as described above. Subsequently, transfection media were removed and replaced with 100 µl of either cPRRSV-2 or HP-PRRSV-2 (equivalent to m.o.i of 1). The cultures were incubated for 1 h (37 °C, humidified 5% CO_2_), then the supernatants were discarded. The cells were then washed twice with 150 µl pre-warmed (37 °C) RPMI^++^ and received 200 µl of pre-warmed RPMI^++^. The cultures were incubated for 48 h (37 °C, humidified 5% CO_2_), then received 50 µl of inducers. The cultures were incubated further for 12 h (37 °C, humidified 5% CO_2_) prior to RNA isolation. Cell culture supernatants (150 µl) were collected for quantification of PRRSV-2 ORF7 RNA by real-time PCR. Controls included MDMs receiving PRRSV-2 and inducers (PRRSV-2-inoculated control); MDMs transfected with Scr1, inoculated with PRRSV-2, and stimulated with inducers (PRRSV-2-inoculated/Scr1 control); and MDMs treated with transfection media alone, inoculated with PRRSV-2, and stimulated with inducers (PRRSV-2-inoculated/transfection media control). MDMs receiving mock Ag plus inducers served as uninoculated control.

PRRSV-2 RNA was isolated and contaminating DNA was eliminated, using Nucleospin^®^ RNA virus kit and rDNase (both from Macherey-Nagel), respectively. The quality and quantity of RNA were evaluated by Nanodrop 2000/2000c spectrophotometer. Reverse transcription (using RevertAid™ First Strand cDNA synthesis kit) and real-time PCR were conducted as described previously [32]. In brief, a total reaction volume of 20 µl, consisting of 2 µl cDNA, 10 µl SYBR^®^ Green PCR master mix (Toyobo), and 400 nM each of primer ORF7 149 F and ORF7 346R was set up in duplicate. The PCR condition was 95 °C (15 min); and 35 cycles of 95 °C (15 s), 53 °C (30 s), and 72 °C (30 s). The C_T_ was collected and compared with the standard curve of C_T_ generated from 10^1^ to 10^8^ copy numbers of recombinant PRRSV-2 ORF7 plasmids. Melting curve analysis and agarose gel electrophoresis were performed to verify a single product. A nuclease-free water was included as no template control in every run.

### Evaluation IFNα protein effects on PRRSV RNA yields in MDMs inoculated with cPRRSV-2 and HP-PRRSV-2

MDMs were treated with 100 µl of rIFNα (Raybiotech, GA) resuspended in pre-warmed RPMI^++^ at 10, 1, and 0.1 ng/ml final. The cultures were incubated for 24 h (37 °C, humidified 5% CO_2_), then received 100 µl of either cPRRSV-2 or HP-PRRSV-2 (equivalent to m.o.i of 1). The cultures were incubated for 1 h (37 °C, humidified 5% CO_2_), then washed and received 200 µl of pre-warmed RPMI^++^ and 50 µl of inducers. The cultures were incubated further for 12 h (37 °C, humidified 5% CO_2_) prior to RNA isolation. Cell culture supernatants (150 µl) were collected for quantification of PRRSV-2 ORF7 RNA by real-time PCR. Controls included MDMs receiving PRRSV-2 and inducers (PRRSV-2-inoculated control), and MDMs receiving mock Ag plus inducers (uninoculated control). Cell viability was determined at the end of culture periods using trypan blue.

### Effects of TGFβ1 and IFNα on PRRSV RNA yields in MDMs inoculated with cPRRSV-2 and HP-PRRSV-2

MDMs were treated with 100 µl of rTGFβ1 (Raybiotech) resuspended in pre-warmed RPMI^++^ at 10 ng/ml final. The cultures were incubated for 24 h (37 °C, humidified 5% CO_2_) prior to receiving 50 µl of rIFNα (10 ng/ml final). The cultures were incubated for 24 h (37 °C, humidified 5% CO_2_), then received 100 µl of either cPRRSV-2 or HP-PRRSV-2 (equivalent to m.o.i of 1). The cultures were incubated for 1 h (37 °C, humidified 5% CO_2_), then the supernatants were removed and the cells were washed and received 200 µl of pre-warmed RPMI^++^ and 50 µl of inducers. The cultures were incubated further for 12 h (37 °C, humidified 5% CO_2_) prior to RNA isolation. Cell culture supernatants (150 µl) were collected for quantification of PRRSV-2 ORF7 RNA by real-time PCR. Controls included MDMs receiving PRRSV-2 and inducers (PRRSV-2-inoculated control); MDMs receiving rTGFβ1, PRRSV-2, and inducers (rTGFβ1-treated/PRRSV-2-inoculated control); MDMs receiving rIFNα, PRRSV-2, and inducers (rIFNα -treated/PRRSV-2-inoculated control); and MDMs receiving mock Ag plus inducers (uninoculated control). Cell viability was determined at the end of culture periods using trypan blue.

### Statistical analysis

Statistical analyses were performed using the SPSS software version 17 (IBM, Armonk, NY). Mean differences of immune-related gene expression levels and TGFβ1 protein levels among groups were tested by one-way ANOVA, followed by Tukey HSD test. Mean differences of percentages of fluoresced cells and PRRSV-2 ORF7 RNA copy numbers among groups were tested by one-way repeated measures ANOVA, followed by Tukey HSD test. P < 0.05 was set as a statistically significant level.

## Results

### TGFβAS1 efficiently knockdown TGFβ1 mRNA expression

Using fluorescent-labeled siRNA control, 1.5% (v/v) transfection media and a transfection period of 4 h yielded the highest transfection efficiency with approximately 60% fluorescent-positive MDMs (Fig. 1B and C). These conditions were therefore used for subsequent transfection experiments.

Phosphorotioate-modified AS ODNs were designed to target various regions of porcine TGFβ1 mRNA (Table 1). Activity-decreasing motifs, i.e., GGGG, ACTG, AAA, TAA, and CCGG were absent from all AS ODNs to ensure the antisense activity. Using optimized transfection conditions, transfection with TGFβAS1, which targeted the AUG region of porcine TGFβ1 mRNA, efficiently knockdown TGFβ1 mRNA expression compared to positive control (Fig. 1D). No knockdown effect on TGFβ1 mRNA expression was observed in MDMs transfected with TGFβAS2-4, TGFβS1-4, and Scr1-4 (Fig. 1D). The knockdown effect of TGFβAS1 was dose-dependent as the effect was observed more strongly at 2 µM than at 1 and 0.5 µM, respectively (Fig. 1E). Significant reduction of TGFβ1 protein level was also detected in TGFβAS1-transfected MDMs (Fig. 1F).

### TGFβAS1 was specific to TGFβ1 mRNA and did not knockdown mRNA expression of any other immune-related genes

The specificity of TGFβAS1 was evaluated by analyzing with BLAST search for its potential target to porcine immune-related genes other than TGFβ1. The TGFβAS1 had no aligned target in any of the immune-related genes presented in this study or essential genes involved in swine immune system (data not shown). The TGFβAS1 also had no aligned target in any ORFs of cPRRSV-2 and HP-PRRSV-2 used in this study (data not shown).

**Fig. 2:**
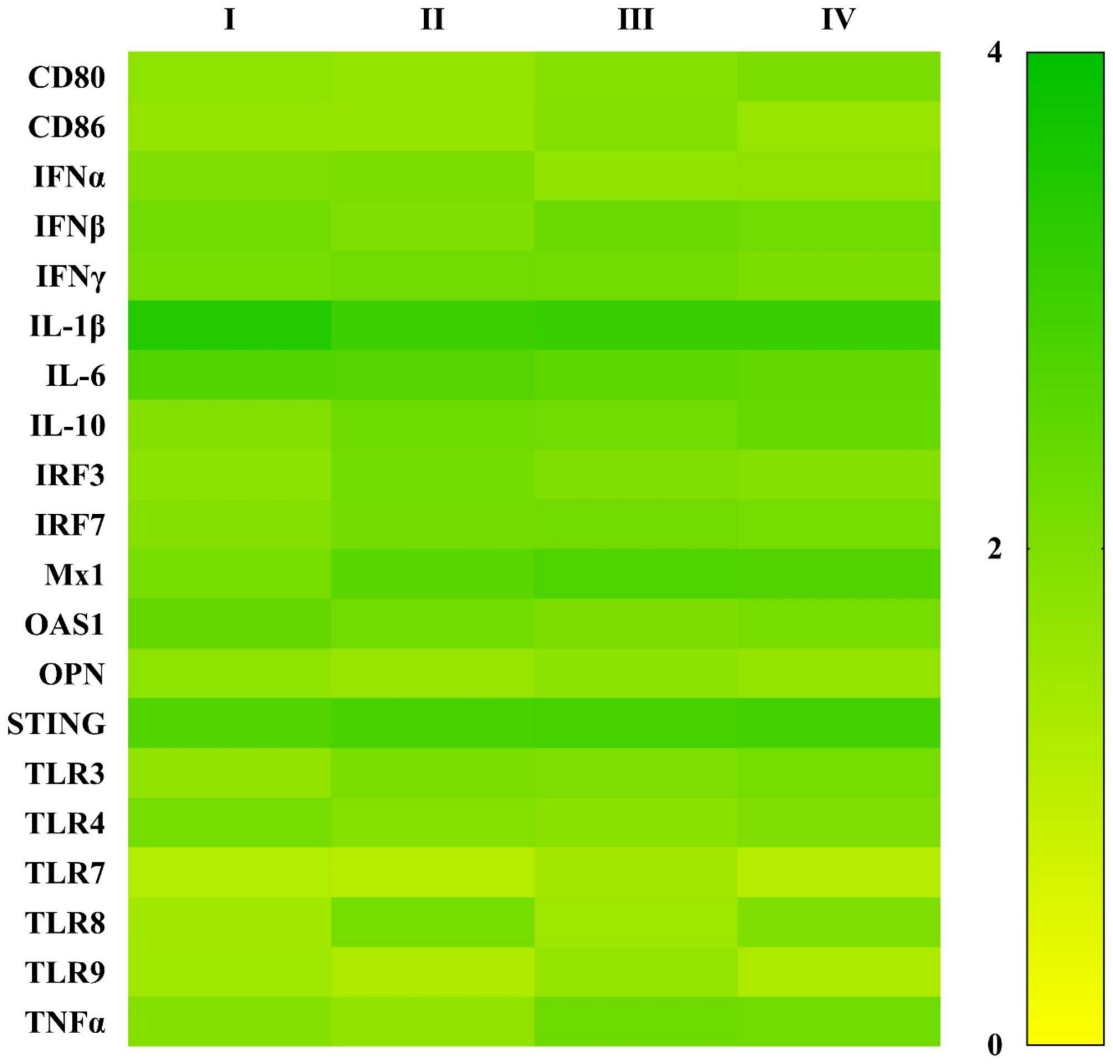
Heat map illustrating expression levels of immune-related genes in MDMs transfected with either TGFβAS1 (I) or Scr1 (II), or otherwise treated with transfection media (III) alone prior to stimulation with a mixture of poly I:C and LPS. Untransfected MDMs stimulated with a mixture of poly I:C and LPS served as positive control (IV). Data were normalized to the geometric average of RPL32 and YWHAZ relative to untransfected/unstimulated MDMs. Data are presented in log 2 scale of “fold” according to 2^(-ΔΔC_T_) method

Compared to positive control, MDMs transfected with TGFβAS1 demonstrated slightly increased mRNA expressions of IFNα (2.0 ± 0.1 vs. 1.7 ± 0.1) and IL-1β (3.4 ± 0.1 vs. 3.1 ± 0.1) (Fig. 2 and Supplementary Table 2). On the other hand, slightly reduced mRNA expressions of IL-10 (1.9 ± 0.1 vs. 2.4 ± 0.1), IRF7 (1.9 ± 0.0 vs. 2.1 ± 0.1), Mx1 (2.1 ± 0.1 vs. 2.7 ± 0.1), TLR3 (1.7 ± 0.0 vs. 2.2 ± 0.1), TLR8 (1.4 ± 0.3 vs. 2.0 ± 0.1), and TNFα (1.9 ± 0.1 vs. 2.2 ± 0.1) were observed. The changes in mRNA expression levels of these genes were not statistically significant, which indicated that TGFβAS1 was specific to TGFβ1 mRNA. TGFβAS1 targeted the AUG region of TGFβ1 mRNA and it can conclude that it does not hybridize to any immune-related genes of interest. MDMs transfected with Scr ODNs or treated with transfection media alone showed no change in mRNA expression level in any immune-related gene as compared to positive control.

### TGFβAS1 significantly knockdown TGFβ1 mRNA expression which was up-regulated by cPRRSV-2 and HP-PRRSV-2 and contributed to improving gene expressions of co-stimulatory molecules, type I IFN, IRGs, and pro-inflammatory cytokine which were down-regulated by the viruses

**Fig. 3:**
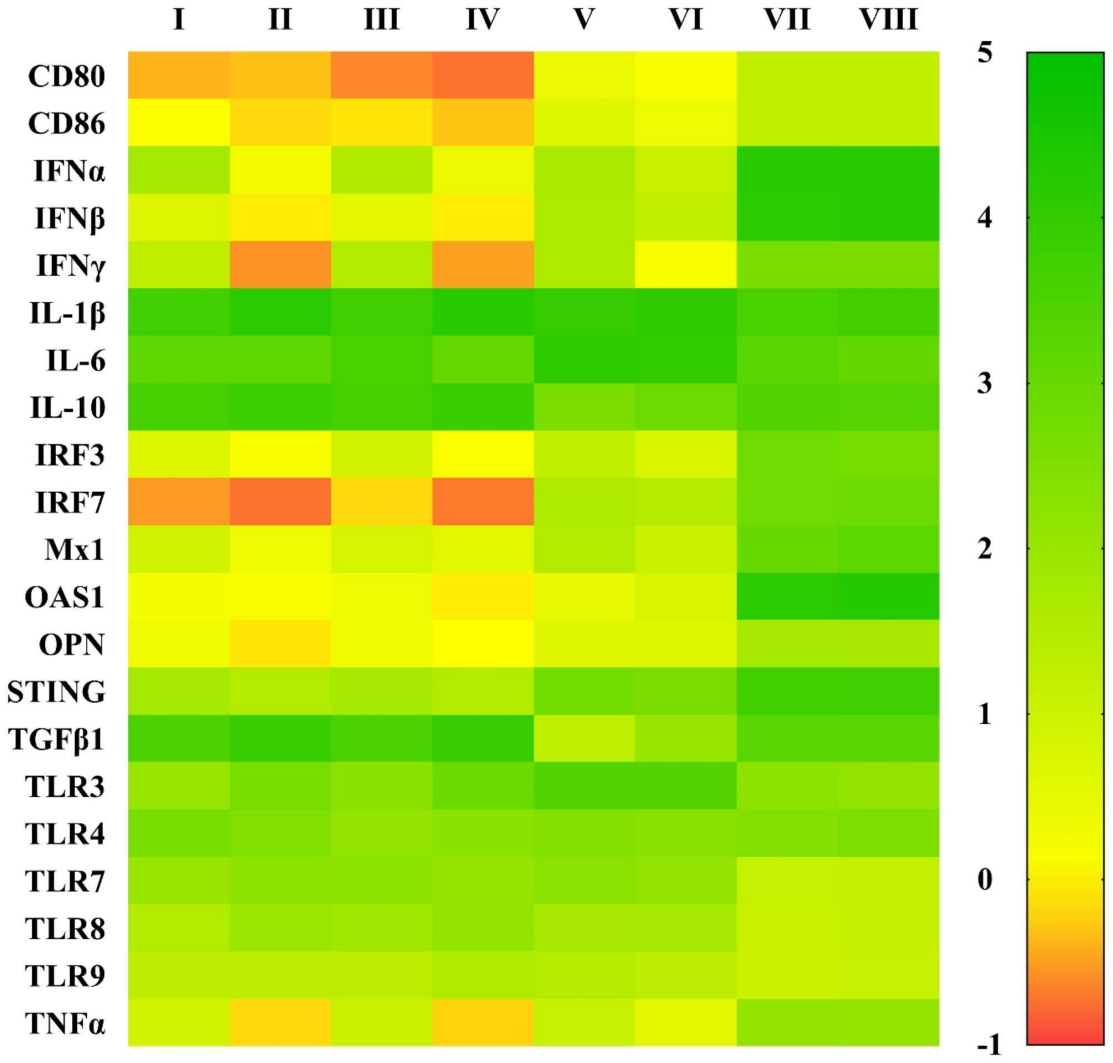
Heat map illustrating effects of TGFβAS1 on immune-related gene expressions in PRRSV-2-inoculated MDMs. MDMs were transfected with TGFβAS1, then inoculated with either cPRRSV-2 or HP-PRRSV-2 and stimulated with a mixture of poly I:C and LPS. MDMs inoculated with cPRRSV-2 or HP-PRRSV-2 and stimulated with a mixture of poly I:C and LPS served as PRRSV-2-inoculated control. MDMs treated with transfection media (Tr. media) and inoculated with cPRRSV-2 or HP-PRRSV-2, then stimulated with a mixture of poly I:C and LPS served as PRRSV-2-inoculated/Tr. media control. MDMs inoculated with mock Ag and stimulated with a mixture of poly I:C and LPS served as mock control. Untreated MDMs stimulated with a mixture of poly I:C and LPS served as positive control (Pos Ctrl). I = cPRRSV-2; II = HP-PRRSV-2; III = Tr. media + cPRRSV-2; IV = Tr. media + HP-PRRSV-2; V = TGFβAS1 + cPRRSV-2; VI = TGFβAS1 + HP-PRRSV-2; VII = Mock Ag; VIII = Pos Ctrl. Data were normalized to the geometric average of RPL32 and YWHAZ relative to untransfected/unstimulated MDMs. Data are presented in log 2 scale of “fold” according to 2^(-ΔΔC_T_) method

Compared to positive control, MDMs inoculated with cPRRSV-2 and HP-PRRSV-2 demonstrated significantly increased TGFβ1, TLR7, and TLR8 mRNA expressions, and significantly reduced CD80, CD86, IFNα, IFNβ, IFNγ, IRF3, IRF7, Mx1, OAS1, OPN, STING, and TNFα mRNA expressions (Fig. 3 and Supplementary Table 3). MDMs inoculated with HP-PRRSV-2 also demonstrated significantly increased IL-1β and TLR3 mRNA expressions. MDMs inoculated with HP-PRRSV-2 showed significantly higher mRNA expressions of TGFβ1, TLR3, and TLR8, and significantly lower mRNA expressions of IFNα, IFNβ, IFNγ, IRF3, Mx1, OPN, and TNFα than MDMs inoculated with cPRRSV-2. Mock Ag had no effect on the modulation of mRNA expressions of these immune-related genes.

**Fig. 4:**
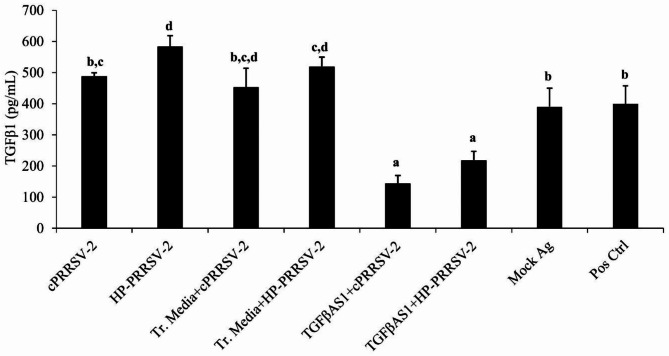
Effect of TGFβAS1 on TGFβ1 protein translation in PRRSV-2-inoculated MDMs. MDMs were transfected with TGFβAS1, then inoculated with either cPRRSV-2 or HP-PRRSV-2, and stimulated with a mixture of poly I:C and LPS. MDMs inoculated with cPRRSV-2 or HP-PRRSV-2 and stimulated with a mixture of poly I:C and LPS served as PRRSV-2-inoculated control. MDMs treated with transfection media (Tr. media) and inoculated with cPRRSV-2 or HP-PRRSV-2, then stimulated with a mixture of poly I:C and LPS served as PRRSV-2-inoculated/Tr. media control. MDMs inoculated with mock Ag and stimulated with a mixture of poly I:C and LPS served as mock control. Untreated MDMs receiving culture media in the presence or absence of a mixture of poly I:C and LPS served as positive and negative controls, respectively. Cell culture supernatants were collected for ELISA. Error bars indicate the SD. Mean differences of TGFβ1 protein translation among groups were tested by one-way ANOVA, followed by Tukey HSD test. Different letters indicate significant differences. P < 0.05 was set as a statistically significant level

Compared to cPRRSV-2-inoculated and HP-PRRSV-2-inoculated MDMs, MDMs transfected with TGFβAS1 prior to cPRRSV-2 and HP-PRRSV-2 inoculation significantly down-regulated TGFβ1 mRNA expression (Fig. 3 and Supplementary Table 3) and protein production (Fig. 4). Interestingly, significantly reduced IL-10 mRNA expression was also observed. On the other hand, the cells demonstrated significantly increased CD80, CD86, IFNβ, IRF3, IRF7, Mx1, OPN, STING, TLR3, and TNFα mRNA expressions. In addition, MDMs transfected with TGFβAS1 prior to HP-PRRSV-2 inoculation demonstrated significantly increased IFNα, IFNγ, and OAS1 mRNA expressions, compared to HP-PRRSV-2-inoculated MDMs. Transfection media had no effect on an alteration of mRNA expressions of these immune-related genes.

### TGFβ1 knockdown significantly contributed to the reduced amount of cPRRSV-2 and HP-PRRSV-2 RNA yields

**Fig. 5:**
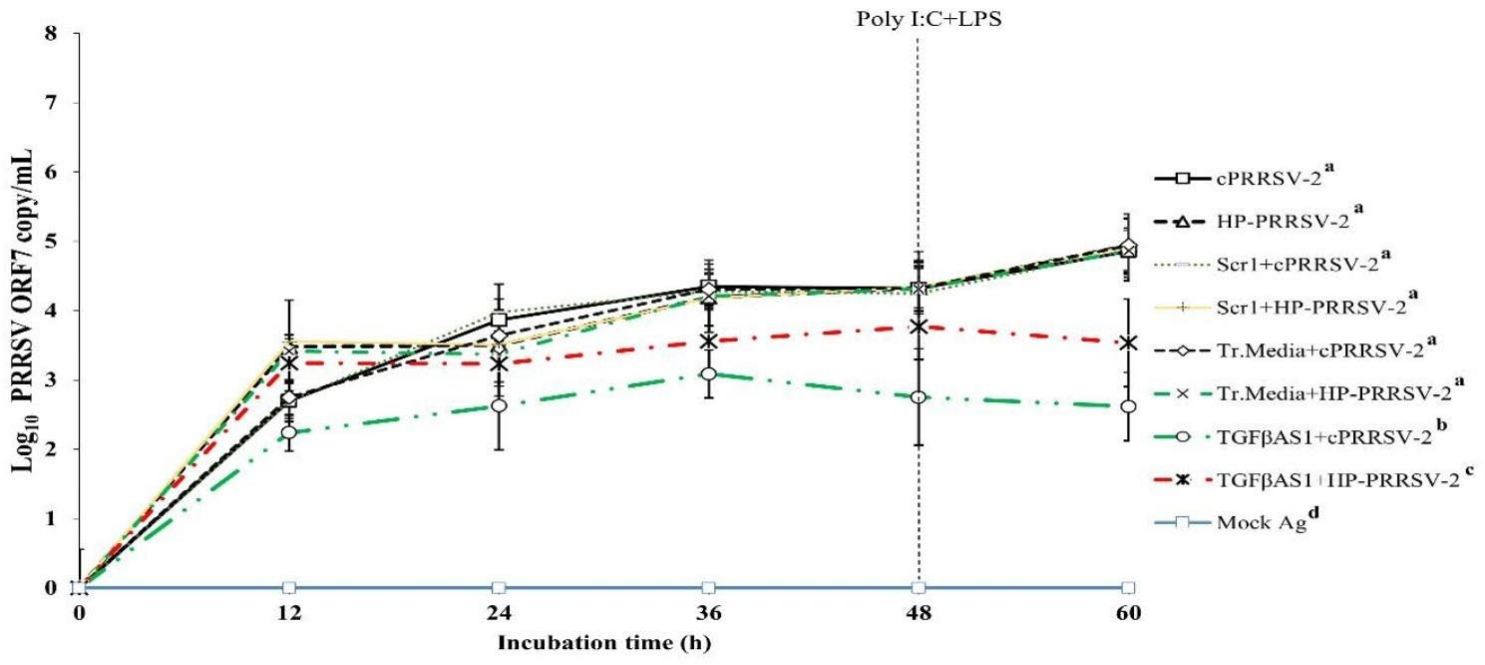
Effect of TGFβ knockdown on PRRSV copy numbers in PRRSV-2-inoculated MDMs. MDMs were transfected with TGFβAS1, then inoculated with either cPRRSV-2 or HP-PRRSV-2 (0 h), and stimulated with a mixture of poly I:C and LPS (48 h). MDMs inoculated with cPRRSV-2 or HP-PRRSV-2 and stimulated with a mixture of poly I:C and LPS served as PRRSV-2-inoculated control. MDMs transfected with Scr1, then inoculated with either cPRRSV-2 or HP-PRRSV-2, and stimulated with a mixture of poly I:C and LPS served as PRRSV-2-inoculated/Scr1 control. MDMs treated with transfection media (Tr. media), then inoculated with cPRRSV-2 or HP-PRRSV-2, and stimulated with a mixture of poly I:C and LPS served as PRRSV-2-inoculated/Tr. media control. MDMs receiving mock Ag plus a mixture of poly I:C and LPS served as uninoculated control. Cell culture supernatants were collected for real-time PCR. The C_T_ values were obtained and PRRSV-2 ORF7 RNA copy numbers were calculated based on the C_T_ standard curve generated from 10^1^-10^8^ copies of recombinant PRRSV-2 ORF7 plasmids. Data were presented in log 10 scale of copy number/ml. Error bars indicate the SD. Mean differences of PRRSV-2 ORF7 RNA copy numbers among groups at time points were tested by one-way repeated measures ANOVA, followed by Tukey HSD. Different superscript letters indicate significant difference. P < 0.05 was set as a statistically significant level

Compared to cPRRSV-2-inoculated MDMs, MDMs transfected with TGFβAS1 prior to cPRRSV-2 inoculation demonstrated significantly lower amount of PRRSV-2 ORF7 RNA copy numbers at 12 (2.2 ± 0.3 vs. 2.7 ± 0.3), 24 (2.6 ± 0.6 vs. 3.9 ± 0.3), 36 (3.1 ± 0.3 vs. 4.3 ± 0.2), 48 (2.8 ± 0.7 vs. 4.3 ± 0.3), and 60 h (2.6 ± 0.5 vs. 4.9 ± 0.3) after inoculation (Fig. 5). Compared to HP-PRRSV-2-inoculated MDMs, MDMs transfected with TGFβAS1 prior to HP-PRRSV-2 inoculation demonstrated significantly lower amount of PRRSV-2 ORF7 RNA copy numbers at 12 (3.2 ± 0.4 vs. 3.5 ± 0.1), 24 (3.2 ± 0.5 vs. 3.5 ± 0.4), 36 (3.6 ± 0.5 vs. 4.2 ± 0.4), 48 (3.8 ± 0.5 vs. 4.3 ± 0.4), and 60 h (3.5 ± 0.6 vs. 4.9 ± 0.4) after inoculation (Fig. 5). Scramble ODNs and transfection media had no effect on an alteration of PRRSV-2 ORF7 RNA copy numbers. No PRRSV-2 ORF7 RNA was detected in MDMs treated with mock Ag.

### IFNα significantly contributed to the reduced amount of cPRRSV-2 and HP-PRRSV-2 RNA yields

Since TGFβ1 knockdown resulted in significantly increased expressions of co-stimulatory molecules, type I IFN, IRGs, TLR3, and TNFα, and significantly reduced amounts of PRRSV-2 ORF7 RNA copy numbers, it is of interest to investigate further the direct effect of those immune-related molecules on PRRSV-2 RNA yields. For such investigation, commercially available rIFNα was chosen as a candidate.

**Fig. 6:**
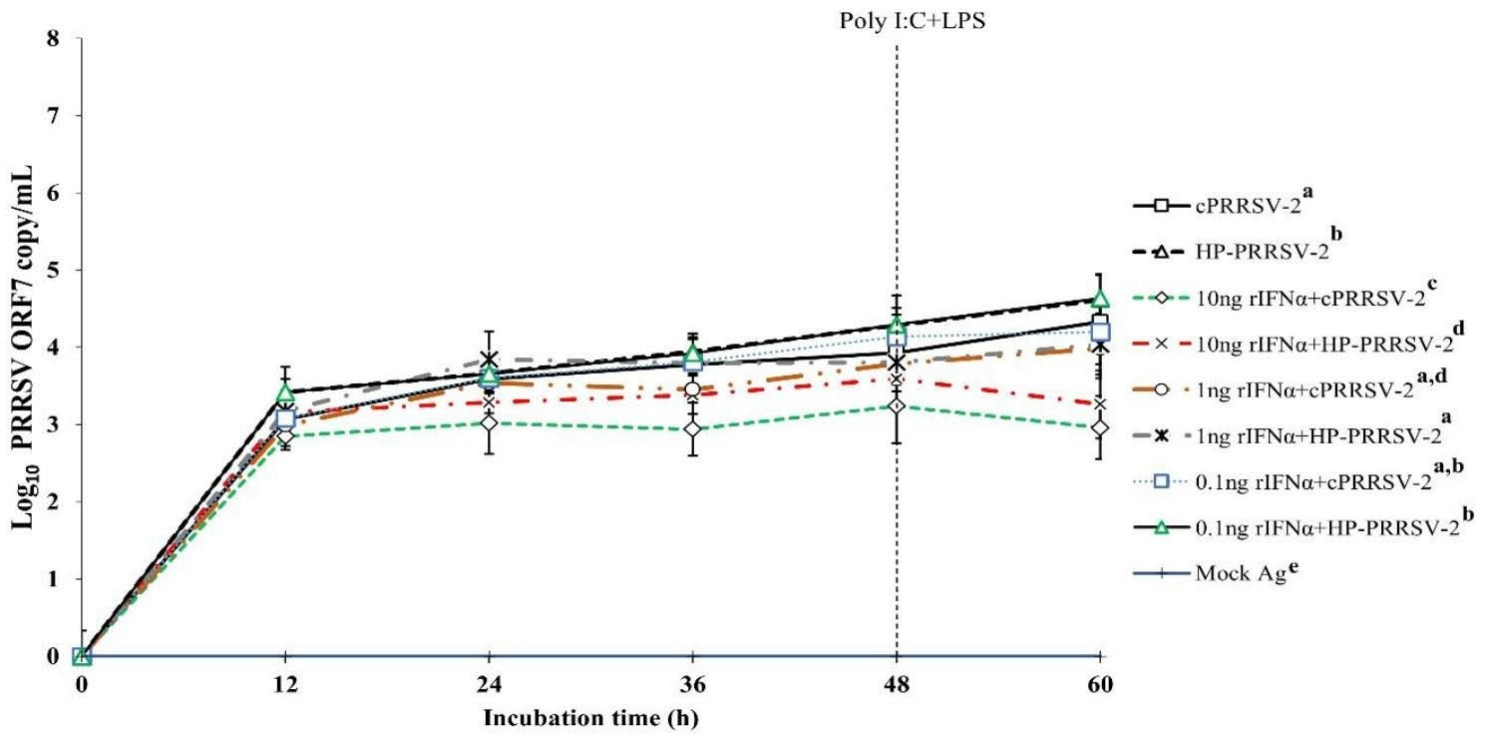
Effects of rIFNα on PRRSV-2 ORF7 RNA copy numbers in PRRSV-2-inoculated MDMs. MDMs were treated with rIFNα (10, 1 and 0.1 ng/ml final), then inoculated with either cPRRSV-2 or HP-PRRSV-2 (0 h), and stimulated with a mixture of poly I:C and LPS (48 h). MDMs inoculated with cPRRSV-2 or HP-PRRSV-2 and stimulated with a mixture of poly I:C and LPS served as PRRSV-2-inoculated control. MDMs receiving mock Ag plus a mixture of poly I:C and LPS served as uninoculated control. Cell culture supernatants were collected for real-time PCR. The C_T_ values were obtained and PRRSV-2 ORF7 RNA copy numbers were calculated based on the C_T_ standard curve generated from 10^1^-10^8^ copies of recombinant PRRSV-2 ORF7 plasmids. Data were presented in log 10 scale of copy number/mL. Error bars indicate the SD. Mean differences of PRRSV-2 ORF7 RNA copy numbers among groups at time points were tested by one-way repeated measures ANOVA, followed by Tukey HSD. Different superscript letters indicate significant differences. P < 0.05 was set as a statistically significant level

Compared to cPRRSV-2-inoculated MDMs, MDMs treated with rIFNα (10 ng/ml final) prior to cPRRSV-2 inoculation demonstrated the significantly lower amount of PRRSV-2 ORF7 RNA copy numbers at 12 (2.9 ± 0.1 vs. 3.1 ± 0.4), 24 (3.0 ± 0.4 vs. 3.6 ± 0.3), 36 (2.9 ± 0.3 vs. 3.8 ± 0.1), 48 (3.2 ± 0.5 vs. 3.9 ± 0.4), and 60 h (3.0 ± 0.4 vs. 4.3 ± 0.3) after inoculation (Fig. 6). MDMs treated with rIFNα (1 and 0.1 ng/ml final) prior to cPRRSV-2 inoculation did not demonstrate lower amount of PRRSV-2 ORF7 RNA copy numbers after inoculation.

Compared to HP-PRRSV-2-inoculated MDMs, MDMs treated with rIFNα (10 ng/ml final) prior to HP-PRRSV-2 inoculation demonstrated significantly lower amount of PRRSV-2 ORF7 RNA copy numbers at 12 (3.2 ± 0.2 vs. 3.4 ± 0.3), 24 (3.3 ± 0.1 vs. 3.7 ± 0.1), 36 (3.4 ± 0.1 vs. 3.9 ± 0.2), 48 (3.6 ± 0.3 vs. 4.3 ± 0.4), and 60 h (3.3 ± 0.4 vs. 4.6 ± 0.3) after inoculation (Fig. 6). MDMs treated with rIFNα (1 ng/ml final) prior to HP-PRRSV-2 inoculation demonstrated significantly lower amount of PRRSV-2 ORF7 RNA copy numbers at 12 (3.2 ± 0.3 vs. 3.4 ± 0.3), 24 (3.8 ± 0.4 vs. 3.7 ± 0.1), 36 (3.8 ± 0.4 vs. 3.9 ± 0.2), 48 (3.8 ± 0.1 vs. 4.3 ± 0.4), and 60 h (4.0 ± 0.4 vs. 4.6 ± 0.3) after inoculation. MDMs treated with rIFNα (0.1 ng/ml final) prior to HP-PRRSV-2 inoculation did not show reduced amount of PRRSV-2 ORF7 RNA copy numbers. No PRRSV-2 ORF7 RNA was detected in MDMs treated with mock Ag.

### TGFβ1 significantly contributed to the increased amount of cPRRSV-2 and HP-PRRSV-2 RNA yields, and decreased the anti-PRRSV effect of IFNα

**Fig. 7:**
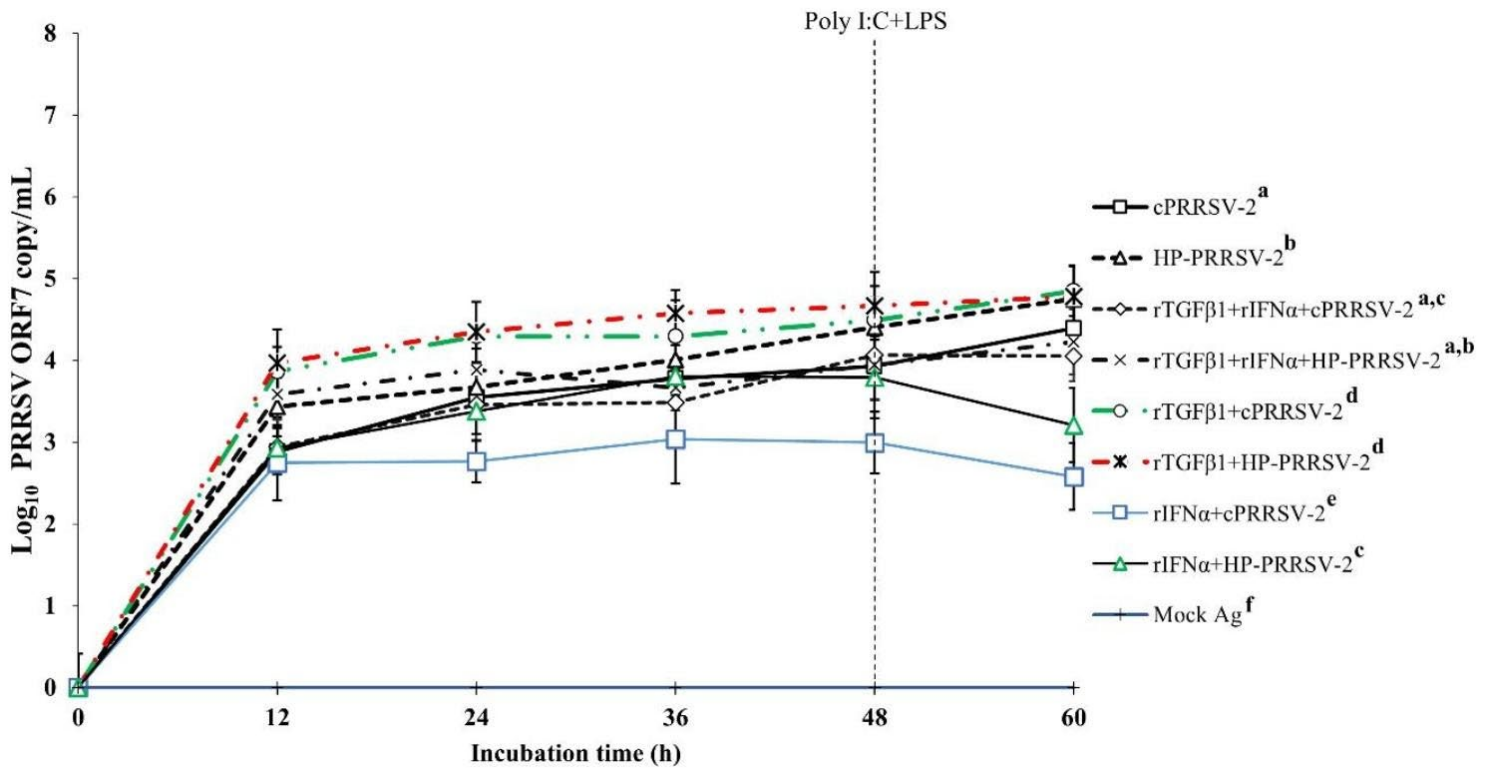
Effects of rTGFβ1 and rIFNα on PRRSV-2 ORF7 RNA copy numbers in PRRSV-2-inoculated MDMs. MDMs were treated with rTGFβ1 (10 ng/ml final), followed by rIFNα (10 ng/ml final), then inoculated with either cPRRSV-2 or HP-PRRSV-2 (0 h), and stimulated with a mixture of poly I:C and LPS (48 h). MDMs inoculated with cPRRSV-2 or HP-PRRSV-2 and stimulated with a mixture of poly I:C and LPS served as PRRSV-2-inoculated control. MDMs treated with rTGFβ1, then inoculated with either cPRRSV-2 or HP-PRRSV-2 (0 h), and stimulated with a mixture of poly I:C and LPS (48 h) served as rTGFβ1-treated/PRRSV-2-inoculated control. MDMs treated with rIFNα, then inoculated with either cPRRSV-2 or HP-PRRSV-2 (0 h), and stimulated with a mixture of poly I:C and LPS (48 h) served as rIFNα -treated/PRRSV-2-inoculated control. MDMs receiving mock Ag plus a mixture of poly I:C and LPS served as uninoculated control. Cell culture supernatants were collected for real-time PCR. The C_T_ values were obtained and PRRSV-2 ORF7 RNA copy numbers were calculated based on the C_T_ standard curve generated from 10^1^-10^8^ copies of recombinant PRRSV-2 ORF7 plasmids. Data were presented in log 10 scale of copy number/mL. Error bars indicate the SD. Mean differences of PRRSV-2 ORF7 RNA copy numbers among groups at time points were tested by one-way repeated measures ANOVA, followed by Tukey HSD. Different superscript letters indicate significant differences. P < 0.05 was set as a statistically significant level

The direct effect of TGFβ1 on PRRSV-2 RNA yields was investigated, together with its effect on anti-PRRSV activity of rIFNα. Compared to cPRRSV-2-inoculated MDMs, MDMs treated with rTGFβ1 prior to cPRRSV-2 inoculation demonstrated significantly higher amount of PRRSV-2 ORF7 RNA copy numbers at 12 (3.9 ± 0.3 vs. 2.9 ± 0.3), 24 (4.3 ± 0.1 vs. 3.5 ± 0.2), 36 (4.3 ± 0.4 vs. 3.8 ± 0.1), 48 (4.5 ± 0.4 vs. 3.9 ± 0.4), and 60 h (4.8 ± 0.3 vs. 4.4 ± 0.2) after inoculation (Fig. 7). Compared to HP-PRRSV-2-inoculated MDMs, MDMs treated with rTGFβ1 prior to HP-PRRSV-2 inoculation demonstrated significantly higher amount of PRRSV-2 ORF7 RNA copy numbers at 12 (4.0 ± 0.4 vs. 3.4 ± 0.4), 24 (4.3 ± 0.4 vs. 3.7 ± 0.3), 36 (4.6 ± 0.3 vs. 4.0 ± 0.3), 48 (4.7 ± 0.4 vs. 4.4 ± 0.4), and 60 h (4.8 ± 0.4 vs. 4.7 ± 0.4) after inoculation (Fig. 7).

Compared to cPRRSV-2-inoculated MDMs, MDMs treated with rIFNα prior to cPRRSV-2 inoculation demonstrated significantly lower amount of PRRSV-2 ORF7 RNA copy numbers at 12 (2.8 ± 0.5 vs. 2.9 ± 0.3), 24 (2.8 ± 0.3 vs. 3.5 ± 0.2), 36 (3.0 ± 0.5 vs. 3.8 ± 0.1), 48 (3.0 ± 0.4 vs. 3.9 ± 0.4), and 60 h (2.6 ± 0.4 vs. 4.4 ± 0.2) after inoculation (Fig. 7). Compared to HP-PRRSV-2-inoculated MDMs, MDMs treated with rIFNα prior to HP-PRRSV-2 inoculation demonstrated significantly lower amount of PRRSV-2 ORF7 RNA copy numbers at 12 (2.9 ± 0.3 vs. 3.4 ± 0.4), 24 (3.4 ± 0.3 vs. 3.7 ± 0.3), 36 (3.8 ± 0.1 vs. 4.0 ± 0.3), 48 (3.8 ± 0.5 vs. 4.4 ± 0.4), and 60 h (3.2 ± 0.5 vs. 4.7 ± 0.4) after inoculation (Fig. 7).

Compared to cPRRSV-2-inoculated MDMs, MDMs treated with rTGFβ1, followed by rIFNα prior to cPRRSV-2 inoculation showed no alteration of the amount of PRRSV-2 ORF7 RNA copy numbers after inoculation (Fig. 7). Likewise, compared to HP-PRRSV-2-inoculated MDMs, MDMs treated with rTGFβ1, followed by rIFNα prior to HP-PRRSV-2 inoculation did not show alteration of the amount of PRRSV-2 ORF7 RNA copy numbers after inoculation (Fig. 7). No PRRSV-2 ORF7 RNA was detected in MDMs treated with mock Ag.

## Discussion

This study investigated the effects of PRRSV-induced TGFβ overexpression on mRNA expressions of co-stimulatory molecules, type I IFN, IRGs, pattern recognition receptors, and pro-inflammatory cytokines in PRRSV-inoculated MDMs. Up-regulation of TGFβ expression has been reported in PRRSV-infected cells, e.g. MDMs and PBMCs, and in lymphoid organs and lungs of PRRSV-infected pigs [18–20]. To date, the role of PRRSV-up-regulated TGFβ expression on immune protection against PRRSV has not yet been studied.

Among all four sequences of phosphorotioate-modified TGFβAS ODNs (Table 1), only those that target AUG region of TGFβ1 mRNA significantly reduced TGFβ1 mRNA expression and protein translation (Fig. 1D-F). In pigs, AUG region has been reported to be a potential target for gene knockdown of at least two cytokines, i.e. IL-10 and IFNγ [33]. The phosphorothioate-modified AS ODNs, theoretically, control target mRNA expression by binding specifically to target mRNA region, and forming mRNA/AS ODN duplexes which then trigger RnaseH to cleave the hybridized target mRNA [34]. This results in reduced amounts or absence of intact mRNA template for translation, and thereby reduced target protein level.

Significantly increased mRNA expressions of TGFβ1, IL-1β, TLR3, TLR7, and TLR8 were detected in MDMs inoculated with cPRRSV-2 and HP-PRRSV-2 (Fig. 3 and Supplementary Table 3). The levels of mRNA expressions of TGFβ1, TLR3, and TLR8 were higher in HP-PRRSV-2-inoculated MDMs than in cPRRSV-2-inoculated MDMs (Fig. 3 and Supplementary Table 3). No change in TLR4 and TLR9 mRNA expression was detected. Similar findings have been reported in pulmonary alveolar macrophages (PAMs) of PRRSV-infected pigs that PAMs from HP-PRRSV-2-infected pigs expressed higher levels of TLR3, TLR7, TLR8, and IL-1β mRNA than PAMs from cPRRSV-2-infected pigs [35]. The up-regulation of TLR3, TLR7, and TLR8 mRNA expression reportedly followed the initial down-regulation of mRNA expression of these genes in PRRSV-infected PAMs and immature DCs [3]. In pigs, up-regulation of TLR3, TLR7, and TLR8 mRNA expression has been reported in lymphoid tissues following PRRSV infection, which was proposedly associated with the increased susceptibility of pigs to secondary infections and the increased severity of the diseases [36].

In contrast to the up-regulation of TGFβ1, TLR7, and TLR8 mRNA expression, both cPRRSV-2 and HP-PRRSV-2 significantly down-regulated mRNA expressions of CD80, CD86, type I and II IFNs (i.e. IFNα, IFNβ, IFNγ), IRGs (i.e. IRF3, IRF7, Mx1, OAS1, OPN, STING), and TNFα in inoculated MDMs (Fig. 3 and Supplementary Table 3). The levels of mRNA expressions of IFNα, IFNβ, IFNγ, IRF3, Mx1, OPN, and TNFα were more reduced in HP-PRRSV-2-inoculated MDMs than in cPRRSV-2-inoculated MDMs (Fig. 3 and Supplementary Table 3). Down-regulation of mRNA expression of CD80 and CD86 has been reported in PRRSV-infected DCs [37]. Suppression of mRNA expression of type I and II IFNs, IRGs, and TNFα has been demonstrated in PBMCs and MDMs inoculated with PRRSV [18, 19, 38]. To date, some mechanisms of PRRSV that mediate a down-regulation of mRNA expression of type I IFN and IRGs have been identified, which include suppression of signaling molecules, i.e. RIG-1, mitochondrial antiviral signaling protein, IRF3, STAT1, ERK, and NFκB [5, 8, 9, 12, 13], degradation of CREB-binding protein [14] and inhibition of nuclear translocation of STAT1 and STAT2 [8]. PRRSV proteins involved in these mechanisms reportedly include Nsp1, Nsp2, Nsp4, Nsp5, Nsp11, GP5, and N protein [5–11].

Transfection of MDMs inoculated with cPRRSV-2 and HP-PRRSV-2 with TGFβAS1 significantly reduced TGFβ1 mRNA expression (Fig. 3 and Supplementary Table 3). Unexpectedly, the transfection with TGFβAS1 also significantly reduced IL-10 mRNA expression of MDMs inoculated with cPRRSV-2 and HP-PRRSV-2 (Fig. 3 and Supplementary Table 3). Significant reduction of IL-10 mRNA expression was not detected in the prior specificity testing of TGFβAS1 in uninoculated MDMs (Fig. 2 and Supplementary Table 2). The reduction percentage of TGFβ1 and IL-10 mRNA expression in TGFβAS1-transfected/cPRRSV-2-inoculated MDMs was approximately 63% and 28%, respectively, whereas that in TGFβAS1-transfected/HP-PRRSV-2-inoculated MDMs was approximately 49% and 26%, respectively. The finding of significantly reduced IL-10 mRNA expression in TGFβAS1-transfected/PRRSV-inoculated MDMs was not clearly understood. TGFβ and IL-10 reportedly promote gene expression of each other [39]. TGFβ secreted by M0 macrophages, together with IL-4 and IL-13, promotes cell differentiation towards IL-10-producing M2 macrophages [40]. Whether or not TGFβ1 knockdown affects PRRSV-mediated M0 macrophage differentiation towards M2 macrophages requires further studies.

In contrast to the significant reduction of TGFβ1 and IL-10 mRNA expression in response to TGFβAS1 transfection, MDMs inoculated with cPRRSV-2 and HP-PRRSV-2 and transfected with TGFβAS1 significantly increased mRNA expressions of CD80, CD86, IFNβ, IRGs (i.e. IRF3, IRF7, Mx1, OPN, STING), TLR3, and TNFα (Fig. 3 and Supplementary Table 3). In addition, transfection of HP-PRRSV-2-inoculated MDMs with TGFβAS1 also significantly increased mRNA expressions of IFNα, IFNγ, and OAS1 (Fig. 3 and Supplementary Table 3). In pigs, little information is available regarding the immunomodulatory activities of TGFβ. The cytokine has been reported to down-regulate CD14, MHCII, IL-6, and TNFα expressions in porcine MDMs [22]. In murine macrophages, TGFβ has been reported to down-regulate CD14, CD40, MHCII and IL-12p40 [23, 24]. In murine T cells, TGFβ reportedly suppresses Th1 cell differentiation, Th1-mediated inflammatory response and expression of IFNγ, IL-2, and IL-4, but promotes Treg differentiation [25]. It should be noted that significantly increased mRNA expressions of immune-related genes in response to TGFβAS1 transfection may have to take the effect of reduced IL-10 expression into account, since IL-10 has been reported to suppress mRNA expressions of these immune-related genes [41], and, in pigs, IL-10 knockdown contributed to significantly increased mRNA expressions of TNFα and IFNγ, and slightly increased mRNA expressions of CD80, CD86, IL-1β, and IL-12p40 [2, 15].

Transfection of MDMs inoculated with cPRRSV-2 and HP-PRRSV-2 with TGFβAS1 significantly reduced PRRSV-2 ORF7 RNA copy numbers (Fig. 5). The reduction was detected from 12 to 60 h after inoculation. The reduction percentage of PRRSV-2 ORF7 RNA copy numbers in TGFβAS1-transfected/cPRRSV-2-inoculated MDMs was approximately 18.5% (at 12 h after inoculation) and 46.9% (at 60 h after inoculation), whereas that in TGFβAS1-transfected/HP-PRRSV-2-inoculated MDMs was approximately 8.6% (at 12 h after inoculation) and 28.6% (at 60 h after inoculation). The reduction of PRRSV-2 ORF7 RNA copy numbers was not due to unspecific binding of TGFβAS1 to PRRSV RNA since there was no aligned target of TGFβAS1 in any ORFs of cPRRSV-2 and HP-PRRSV-2 used in this study. It is noteworthy that the reduction percentage of PRRSV-2 ORF7 RNA copy numbers and TGFβ1 mRNA expression was higher in TGFβAS1-transfected/cPRRSV-2-inoculated MDMs than in TGFβAS1-transfected/HP-PRRSV-2-inoculated MDMs.

In addition to significantly reduced TGFβ1 mRNA expression, significantly reduced PRRSV-2 ORF7 RNA copy numbers were associated with significantly increased mRNA expressions of CD80, CD86, IFNα, IFNβ, IFNγ, IRGs (i.e. IRF3, IRF7, Mx1, OAS1, OPN, STING), TLR3, and TNFα in TGFβAS1-transfected/cPRRSV-2-inoculated MDMs and TGFβAS1-transfected/HP-PRRSV-2-inoculated MDMs. Some of these immune-related genes, i.e. IFNα, IFNβ, IFNγ, and TNFα have been reported for their inhibitory effect against PRRSV replication [42–45]. Overexpression of OAS1, STING, and TLR3 reportedly contributed to decreasing PRRSV replication in MARC-145 cells and PAMs [46–48]. To elucidate the contribution of these immune-related genes in reducing PRRSV-2 ORF7 RNA copy numbers, commercially available rIFNα was used. Treatment of MDMs with optimal concentration of rIFNα prior to either cPRRSV-2 or HP-PRRSV-2 inoculation significantly reduced PRRSV-2 ORF7 RNA copy numbers (Fig. 6). These findings suggest that significantly increased expressions of immune-related genes in response to TGFβ1 knockdown might contribute to the reduction of PRRSV-2 ORF7 RNA copy numbers.

To elucidate further whether PRRSV-up-regulated TGFβ1 expression supports PRRSV replication, rTGFβ1 was used. Treatment of MDMs with rTGFβ1 prior to either cPRRSV-2 or HP-PRRSV-2 inoculation significantly increased PRRSV-2 ORF7 RNA copy numbers (Fig. 7). Treatment of MDMs with rTGFβ1 prior to rIFNα treatment and cPRRSV-2 or HP-PRRSV-2 inoculation reduced the antiviral activity of rIFNα (Fig. 7). These findings clearly indicate the positive role of TGFβ1 on PRRSV replication. These findings also suggest a strategy of PRRSV to enhance virus replication and reduce innate immune defense against the virus through an up-regulation of TGFβ1 expression.

## Conclusion

Both cPRRSV-2 and HP-PRRSV-2 significantly induced TGFβ1 mRNA expression in MDMs. TGFβ1 protein translation in MDMs was significantly induced by HP-PRRSV-2. Knockdown of TGFβ1 expression by TGFβAS1 significantly improved mRNA expression levels of CD80, CD86, IFNβ, IRGs (i.e. IRF3, IRF7, Mx1, OPN, STING), TLR3, and TNFα in MDMs inoculated with the virus. Knockdown of TGFβ1 expression by TGFβAS1 significantly contributed to the reduced yields of PRRSV-2 RNA copy numbers. On the contrary, recombinant TGFβ1 sustained the yields of PRRSV-2 RNA copy numbers. These findings demonstrate a potential innate immune suppressive strategy of PRRSV and the immunomodulatory role of PRRSV-induced TGFβ on downmodulating innate immune defense against the virus. These findings also suggest a potential target that a development of future PRRSV vaccines and vaccine adjuvants should take into consideration.

### Electronic supplementary material

Below is the link to the electronic supplementary material.

Supplementary Material 1

Supplementary Material 2

Supplementary Material 3

Supplementary Material 4

Supplementary Material 5

## Data Availability

The original contributions presented in the study are included in the article/Supplementary material. The datasets used and/or analyzed during the current study are available from the corresponding author on reasonable request.
