# Supplementary material for: Knock down of transforming growth factor beta improves expressions of co-stimulatory molecules, type I interferon-regulated genes, and pro-inflammatory cytokine in PRRSV-inoculated monocyte-derived macrophages
Source: BMC Vet Res. 2024 Aug 3;20:344. doi: 10.1186/s12917-023-03760-8 (PMC11297646; doi:10.1186/s12917-023-03760-8)
Supplement: Supplementary file 1 — Supplementary Material 1 [file 12917_2023_3760_MOESM1_ESM.docx]

**Supplementary Table 1** Primer details and optimized conditions for all genes presented in this study.

| Gene | Primer sequence (5’-3’) | Primer  location | Target sequence | Size  (bp) | Ta^*^  (°C) | Primer  Conc.  (nM) | E^*^  (%) | r^2^ | Tm^*^  (°C) |
| --- | --- | --- | --- | --- | --- | --- | --- | --- | --- |
| CD80^a^ | F: AATGGTCAAAGCTGACTTTCCTG  R: GGTTGAGCACCTTATCCTTTTGA | 712-734  796-774 | NM_214087.1 | 85 | 55 | 300 | 96 | 0.997 | 77 |
| CD86^a^ | F: CCCCTCTAATGAATGTGGTGAAAC  R: GATCGTTCATGGACTTCTGCTCT | 822-845  908-886 | NM_214222.1 | 87 | 55 | 400 | 94 | 1.000 | 77 |
| IFNα^b^ | F: AGCCTCCTGCACCAGTTCTG  R: TCACAGCCAGGATGGAGTCC | 346-365  469-450 | NM_214393.1 | 124 | 60 | 300 | 97 | 0.996 | 86 |
| IFNβ^b^ | F: TAGCACTGGCTGGAATGAAACC  R: TCAGGTGAAGAATGGTCATGTCT | 288-309  427-405 | NM_001003923.1 | 140 | 57 | 300 | 91 | 0.994 | 83 |
| IFNγ^c^ | F: TGGTAGCTCTGGGAAACTGAATG  R: GGCTTTGCGCTGGATCTG | 342-364  420-403 | NM_213948 | 79 | 60 | 400 | 102 | 1.000 | 78 |
| IL-1β^d^ | F: AACGTGCAGTCTATGGAGT  R: GAACACCACTTCTCTCTTCA | 412-430  534-515 | NM_214055.1 | 123 | 55 | 300 | 93 | 0.999 | 82 |
| IL-6^e^ | F: CTGGCAGAAAACAACCTGAACC | 316-337 | NM_214399.1 | 94 | 55 | 300 | 100 | 0.994 | 78 |
|  | R: GATTCTCATCAAGCAGGTCTCC | 409-387 |  |  |  |  |  |  |  |
| IL-10^c^ | F: CGGCGCTGTCATCAATTTCTG  R: CCCCTCTCTTGGAGCTTGCTA | 430-450  518-498 | NM_214041 | 89 | 60 | 200 | 96 | 0.999 | 82 |
| IRF3^g^ | F: CATGGACTTGGCCTCGGATG  R: TTTCACGGACTCCCAGGTTG | 438-457  562-543 | NM_213770.1 | 125 | 60 | 300 | 90 | 0.998 | 88 |
| IRF7^g^ | F: AAAACCAACTTCCGCTGTGC  R: GTTGAAGCCTGGGCCTTCTC | 482-501  619-600 | NM_001097428.1 | 138 | 58 | 300 | 97 | 0.998 | 86 |
| Mx1^b^ | F: AGTGTCGGCTGTTTACCAAG  R: TTCACAAACCCTGGCAACTC | 1302-1321  1452-1433 | NM_214061 | 151 | 57 | 300 | 98 | 0.997 | 80 |
| OAS1^b^ | F: CCCTGTTCGCGTCTCCAAAG  R: GCGGGCAGGACATCAAACTC | 197-216  499-480 | NM_214303.1 | 303 | 60 | 300 | 95 | 0.997 | 86 |
| OPN^b^ | F: TTGGACAGCCAAGAGAAGGACAGT  R: GCTCATTGCTCCCATCATAGGTCTTG | 731-754  851-826 | NM_214023.1 | 121 | 56 | 300 | 102 | 0.998 | 83 |
| STING^d^ | F: TTACATCGGGTACCTGCGGC  R: CCGAGTACGTTCTTGTGGCG | 489-508  572-553 | NM_001142838.1 | 84 | 60 | 300 | 92 | 0.995 | 83 |
| TGFβ1^c^ | F: TACGCCAAGGAGGTCACCC  R: CAGCTCTGCCCGAGAGAGC | 1215-1233  1370-1352 | NM_214015.1 | 156 | 60 | 400 | 101 | 0.991 | 84 |
| TLR3^f^ | F: GCATTGCCTGGTTTGTTAGTTG  R: TGTATCAAAAAGAATCACTGGGAG | 2173-2194  2294-2271 | NM_001097444 | 122 | 55 | 400 | 94 | 0.993 | 82 |
| TLR4^d^ | F: CGAGGCCGTCATTAGT | 2141-2156 | NM_001113039.2 | 144 | 55 | 300 | 91 | 0.985 | 81 |
|  | R: ACAAAGGCGTCATAGGT | 2284-2268 |  |  |  |  |  |  |  |
| TLR7^f^ | F: TTTCCTCAAATGCCTAAACTTAT | 1560-1582 | NM_001097434.1 | 185 | 55 | 400 | 91 | 1.000 | 81 |
|  | R: AGTGGCTGTTACTACTTATATCT | 1744-1722 |  |  |  |  |  |  |  |
| TLR8^d^ | F: GCATTGCCTGGTTTGTTAGTTG | 2173-2194 | NM_001097444 | 122 | 58 | 400 | 98 | 0.992 | 77 |
|  | R: TGTATCAAAAAGAATCACTGGGAG | 2294-2271 |  |  |  |  |  |  |  |
| TLR9^h^ | F: GCCATTACTAGGGAGGTGGA | 1354-1373 | NM_213958.1 | 111 | 58 | 500 | 95 | 0.996 | 83 |
|  | R: CTTGCAGTTTGGCATGAAGT | 1464-1445 |  |  |  |  |  |  |  |
| TNFα^c^ | F: AGCCTCTTCTCCTTCCTCCTG  R: GAGACGATGATCTGAGTCCTTGG | 283-303  427-405 | NM_214022 | 145 | 60 | 300 | 93 | 0.994 | 84 |
| RPL32^b^ | F: CGGAAGTTTCTGGTACACAATGTAA  R: TGGAAGAGACGTTGTGAGCAA | 249-273  342-322 | NM_001001636.1 | 94 | 55 | 400 | 99 | 0.993 | 79 |
| YWHAZ^b^ | F: ATTGGGTCTGGCCCTTAACT  R: GCGTGCTGTCTTTGTATGACTC | 961-980  1106-1085 | XM_001927228.4 | 146 | 58 | 300 | 91 | 0.998 | 80 |

^*^Ta, Annealing temperature; E, Efficiency from serial dilutions of reference cDNA; Tm, Melting temperature

^a^ Volf, J., Stepanova, H., Matiasovic, J., Kyrova, K., Sisak, F., Havlickova, H., Leva, L., Faldyna, M., and Rychlik, I. (2012). Salmonella enterica serovar Typhimurium and Enteritidis infection of pigs and cytokine signalling in palatine tonsils. *Vet Microbiol* 156**,** 127-135.

^b^ Fossum, C., Hjertner, B., Ahlberg, V., Charerntantanakul, W., Mcintosh, K., Fuxler, L., Balagunaseelan, N., Wallgren, P., and Lovgren Bengtsson, K. (2014). Early inflammatory response to the saponin adjuvant Matrix-M in the pig. *Vet Immunol Immunopathol* 158**,** 53-61.

^c^ Wikstrom, F.H., Fossum, C., Fuxler, L., Kruse, R., and Lovgren, T. (2011). Cytokine induction by immunostimulatory DNA in porcine PBMC is impaired by a hairpin forming sequence motif from the genome of Porcine Circovirus type 2 (PCV2). *Vet Immunol Immunopathol* 139**,** 156-166.

^d^ Tu, P.Y., Tsai, P.C., Lin, Y.H., Liu, P.C., Chang, H.L., Kuo, T.Y., and Chung, W.B. (2015). Expression profile of Toll-like receptor mRNA in pigs co-infected with porcine reproductive and respiratory syndrome virus and porcine circovirus type 2. *Res Vet Sci* 98**,** 134-141.

^e^ Duvigneau, J.C., Hartl, R.T., Groiss, S., and Gemeiner, M. (2005). Quantitative simultaneous multiplex real-time PCR for the detection of porcine cytokines. *J Immunol Methods* 306, 16-27.

^f^ Chen, Y., Wang, Y., Zeng, K., Lei, Y.F., Chen, X.H., and Ying, S.C. (2018). Knockdown expression of IL-10Rα gene inhibits PRRSV replication and elevates immune responses in PBMCs of Tibetan pig in vitro. *Vet Res Communications* 42, 11-18.

^g^ Tu, P.Y., Tsai, P.C., Lin, Y.H., Chang, H.L., Kuo, T.Y., and Chiou, M.T. (2015). Expression of Toll-like receptor signaling-related genes in pigs co-infected with porcine reproductive and respiratory syndrome virus and porcine circovirus type 2. *Res Vet Sci* 101, 180-186.

^h^ Kuzemtseva, L., de la Torre, E., Martin, G., Soldevila, F., Ait-Ali, T., and Mateu, E. (2014). Regulation of toll-like receptors 3, 7 and 9 in porcine alveolar macrophages by different genotype 1 strains of porcine reproductive and respiratory syndrome virus. *Vet Immunol Immunopathol* 158, 189-198.
