# Supplementary material for: Knock down of transforming growth factor beta improves expressions of co-stimulatory molecules, type I interferon-regulated genes, and pro-inflammatory cytokine in PRRSV-inoculated monocyte-derived macrophages
Source: BMC Vet Res. 2024 Aug 3;20:344. doi: 10.1186/s12917-023-03760-8 (PMC11297646; doi:10.1186/s12917-023-03760-8)
Supplement: Supplementary file 2 — Supplementary Material 2 [file 12917_2023_3760_MOESM2_ESM.docx]

**Supplementary Table 2** Expression levels of immune-related genes in MDMs (*n* =8 pigs) transfected with either TGFβAS1 or Scr1, or otherwise treated with transfection media (Tr. media) alone prior to stimulation with a mixture of poly I:C and LPS.

| Gene | TGFβAS1 | Scr1 | Tr. media | Pos Ctrl |
| --- | --- | --- | --- | --- |
| CD80 | 1.8 ± 0.1 | 1.7 ± 0.1 | 1.9 ± 0.2 | 2.0 ± 0.2 |
| CD86 | 1.7 ± 0.2 | 1.6 ± 0.2 | 1.9 ± 0.2 | 1.6 ± 0.2 |
| IFNα | 2.0 ± 0.1 | 2.0 ± 0.2 | 1.7 ± 0.0 | 1.7 ± 0.1 |
| IFNβ | 2.2 ± 0.1 | 2.0 ± 0.0 | 2.3 ± 0.1 | 2.2 ± 0.1 |
| IFNγ | 2.1 ± 0.1 | 2.2 ± 0.3 | 2.2 ± 0.0 | 2.0 ± 0.1 |
| IL-1β | 3.4 ± 0.1 | 3.0 ± 0.3 | 3.1 ± 0.0 | 3.1 ± 0.1 |
| IL-6 | 2.7 ± 0.1 | 2.7 ± 0.2 | 2.5 ± 0.1 | 2.5 ± 0.1 |
| IL-10 | 1.9 ± 0.1 | 2.3 ± 0.2 | 2.2 ± 0.1 | 2.4 ± 0.1 |
| IRF3 | 1.8 ± 0.0 | 2.2 ± 0.3 | 2.0 ± 0.2 | 1.9 ± 0.1 |
| IRF7 | 1.9 ± 0.0 | 2.2 ± 0.2 | 2.1 ± 0.2 | 2.1 ± 0.1 |
| Mx1 | 2.1 ± 0.1 | 2.6 ± 0.3 | 2.8 ± 0.1 | 2.7 ± 0.1 |
| OAS1 | 2.4 ± 0.3 | 2.2 ± 0.0 | 2.0 ± 0.2 | 2.1 ± 0.1 |
| OPN | 1.7 ± 0.2 | 1.6 ± 0.0 | 1.8 ± 0.1 | 1.7 ± 0.1 |
| STING | 2.7 ± 0.1 | 2.8 ± 0.1 | 2.9 ± 0.1 | 2.9 ± 0.1 |
| TLR3 | 1.7 ± 0.0 | 2.0 ± 0.2 | 2.0 ± 0.0 | 2.2 ± 0.1 |
| TLR4 | 2.1 ± 0.2 | 1.9 ± 0.1 | 1.9 ± 0.1 | 2.0 ± 0.1 |
| TLR7 | 1.2 ± 0.0 | 1.1 ± 0.1 | 1.4 ± 0.1 | 1.1 ± 0.1 |
| TLR8 | 1.4 ± 0.3 | 2.1 ± 0.1 | 1.5 ± 0.3 | 2.0 ± 0.1 |
| TLR9 | 1.5 ± 0.3 | 1.3 ± 0.1 | 1.7 ± 0.2 | 1.3 ± 0.1 |
| TNFα | 1.9 ± 0.1 | 1.7 ± 0.2 | 2.3 ± 0.1 | 2.2 ± 0.1 |
| Data were normalized to the geometric average of RPL32 and YWHAZ in relative to untransfected/unstimulated MDMs. Data are presented in log 2 scale of “fold” according to 2^(-ΔΔC_T_) method (Mean + SD). | | | | |
