# Supplementary material for: Knock down of transforming growth factor beta improves expressions of co-stimulatory molecules, type I interferon-regulated genes, and pro-inflammatory cytokine in PRRSV-inoculated monocyte-derived macrophages
Source: BMC Vet Res. 2024 Aug 3;20:344. doi: 10.1186/s12917-023-03760-8 (PMC11297646; doi:10.1186/s12917-023-03760-8)
Supplement: Supplementary file 3 — Supplementary Material 3 [file 12917_2023_3760_MOESM3_ESM.docx]

**Supplementary Table 3** Effects of TGFβAS1 transfection on expression levels of immune-related genes in MDMs (*n* = 8 pigs) inoculated with either cPRRSV-2 or HP-PRRSV-2 and stimulated with a mixture of poly I:C and LPS.

| **Gene** | **cPRRSV-2-inoculated Ctrl** | **HP-PRRSV-2-inoculated Ctrl** | **cPRRSV-2-inoculated transfection media Ctrl** | **HP-PRRSV-2-inoculated/ transfection media Ctrl** | **cPRRSV-2-inoculated/ TGFβAS1-transfected** | **HP-PRRSV-2-inoculated/ TGFβAS1-transfected** | **Mock**  **Ctrl** | **Pos**  **Ctrl** |  |
| --- | --- | --- | --- | --- | --- | --- | --- | --- | --- |
|  |  |  |  |  |  |  |  |  |  |
| CD80 | -0.4 ± 0.1^a^ | -0.4 ± 0.2^a^ | -0.6 ± 0.0^a,b^ | -0.7 ± 0.0^b^ | 0.4 ± 0.2^c^ | 0.1 ± 0.0^d^ | 1.2 ± 0.1^e^ | 1.2 ± 0.2^e^ |  |
| CD86 | 0.0 ± 0.1^a,c^ | -0.2 ± 0.2^a^ | -0.1 ± 0.0^a^ | -0.3 ± 0.0^a^ | 0.6 ± 0.0^b^ | 0.3 ± 0.1^b,c^ | 1.2 ± 0.1^d^ | 1.2 ± 0.0^d^ |  |
| IFNα | 1.7 ± 0.3^a^ | 0.2 ± 0.1^b^ | 1.5 ± 0.0^c^ | 0.3 ± 0.0^b^ | 1.7 ± 0.1^a^ | 1.1 ± 0.1^d^ | 4.2 ± 0.4^e^ | 4.2 ± 0.3^e^ |  |
| IFNβ | 0.7 ± 0.2^a^ | -0.1 ± 0.2^b^ | 0.5 ± 0.0^a^ | -0.1 ± 0.0^b^ | 1.6 ± 0.2^c^ | 1.2 ± 0.1^d^ | 4.1 ± 0.2^e^ | 4.2 ± 0.3^e^ |  |
| IFNγ | 1.3 ± 0.2^a^ | -0.6 ± 0.3^b^ | 1.5 ± 0.3^a^ | -0.5 ± 0.1^b^ | 1.6 ± 0.1^a^ | 0.1 ± 0.1^c^ | 2.6 ± 0.1^d^ | 2.6 ± 0.1^d^ |  |
| IL-1β | 3.7 ± 0.1^a,b^ | 4.1 ± 0.2^b,c^ | 3.7 ± 0.1^a^ | 4.2 ± 0.4^c^ | 3.9 ± 0.3^a,b,c^ | 4.1 ± 0.2^b,c^ | 3.6 ± 0.2^a^ | 3.6 ± 0.4^a^ |  |
| IL-6 | 3.1 ± 0.4^a^ | 3.1 ± 0.3^a^ | 3.5 ± 0.4^a^ | 3.0 ± 0.1^a^ | 4.1 ± 0.2^a^ | 4.0 ± 0.2^a^ | 3.2 ± 0.3^a^ | 3.1 ± 0.3^a^ |  |
| IL-10 | 3.6 ± 0.3^a,b^ | 3.8 ± 0.1^a,b^ | 3.6 ± 0.3^a^ | 3.8 ± 0.3^a^ | 2.6 ± 0.2^c^ | 2.8 ± 0.1^c^ | 3.4 ± 0.1^b^ | 3.3 ± 0.2^b^ |  |
| IRF3 | 0.6 ± 0.2^a^ | 0.1 ± 0.2^b^ | 0.9 ± 0.1^c^ | 0.1 ± 0.0^b^ | 1.2 ± 0.3^d^ | 0.7 ± 0.1^a^ | 2.8 ± 0.2^e^ | 2.7 ± 0.3^f^ |  |
| IRF7 | -0.5 ± 0.1^a,b^ | -0.7 ± 0.1^a^ | -0.2 ± 0.0^b^ | -0.7 ± 0.0^a^ | 1.6 ± 0.3^c^ | 1.4 ± 0.1^c^ | 2.8 ± 0.2^d^ | 2.8 ± 0.1^d^ |  |
| Mx1 | 0.9 ± 0.2^a,c^ | 0.3 ± 0.3^b^ | 0.8 ± 0.1^a,c^ | 0.5 ± 0.0^a,b^ | 1.5 ± 0.3^d^ | 1.0 ± 0.1^c^ | 3.0 ± 0.2^e^ | 3.1 ± 0.3^e^ |  |
| OAS1 | 0.2 ± 0.1^a,c^ | 0.1 ± 0.2^a,b^ | 0.3 ± 0.0^a,c^ | -0.1 ± 0.0^b^ | 0.4 ± 0.2^c^ | 0.7 ± 0.1^d^ | 4.1 ± 0.1^e^ | 4.2 ± 0.1^e^ |  |
| OPN | 0.3 ± 0.2^a^ | -0.1 ± 0.0^b^ | 0.3 ± 0.1^a^ | 0.0 ± 0.1^b^ | 0.6 ± 0.1^c^ | 0.7 ± 0.2^c^ | 1.7 ± 0.0^d^ | 1.7 ± 0.3^d^ |  |
| STING | 1.7 ± 0.0^a,b^ | 1.5 ± 0.0^a^ | 1.7 ± 0.2^b^ | 1.5 ± 0.0^a,b^ | 2.7± 0.2^c^ | 2.5 ± 0.3^c^ | 3.7 ± 0.2^d^ | 3.7 ± 0.0^d^ |  |
| TGFβ1 | 3.5 ± 0.1^a^ | 3.9 ± 0.2^b^ | 3.5 ± 0.2^a^ | 3.9 ± 0.0^b^ | 1.3 ± 0.2^c^ | 2.0 ± 0.1^d^ | 3.2 ± 0.2^e^ | 3.2 ± 0.1^e^ |  |
| TLR3 | 2.0 ± 0.2^a^ | 2.6 ± 0.0^b,c^ | 2.3 ± 0.2^a,b^ | 2.9 ± 0.1^c^ | 3.4 ± 0.1^d^ | 3.4 ± 0.2^d^ | 2.2 ± 0.1^a,b^ | 2.1 ± 0.2^a^ |  |
| TLR4 | 2.6 ± 0.0^a^ | 2.4 ± 0.2^a,b^ | 2.1 ± 0.1^b^ | 2.3 ± 0.1^a,b^ | 2.4 ± 0.3^a,b^ | 2.3 ± 0.2^a,b^ | 2.4 ± 0.1^a,b^ | 2.5 ± 0.0^a^ |  |
| TLR7 | 2.0 ± 0.0^a^ | 2.2 ±0.1^a^ | 2.2 ± 0.2^a^ | 2.1 ± 0.1^a^ | 2.2 ± 0.2^a^ | 2.1 ± 0.2^a^ | 1.1 ± 0.1^b^ | 1.1 ± 0.1^b^ |  |
| TLR8 | 1.5 ± 0.1^a^ | 1.9 ± 0.2^b,c^ | 1.8 ± 0.1^a,b,c^ | 2.1 ± 0.1^b^ | 1.7 ± 0.2^a,c^ | 1.7 ± 0.2^a,c^ | 1.1 ± 0.1^d^ | 1.1 ± 0.3^d^ |  |
| TLR9 | 1.3 ± 0.1^a,b^ | 1.3 ± 0.1^a,b^ | 1.3 ± 0.1^a,b^ | 1.5 ± 0.0^a^ | 1.4 ± 0.1^a^ | 1.3 ± 0.1^a,b^ | 1.0 ± 0.1^b^ | 1.1 ± 0.3^b^ |  |
| TNFα | 0.9 ± 0.2^a,b^ | -0.2 ± 0.1^c^ | 1.0 ± 0.2^b,d^ | -0.2 ± 0.0^c^ | 1.1 ± 0.2^d^ | 0.5 ± 0.1^a^ | 2.1 ± 0.1^e^ | 2.1 ± 0.1^e^ |  |

Data were normalized to the geometric average of RPL32 and YWHAZ in relative to untransfected/unstimulated MDMs. Data are presented in log 2 scale of “fold” according to 2^(-ΔΔC_T_) method (Mean + SD). Mean differences of immune-related gene expression levels among groups were tested by one-way ANOVA, followed by Tukey HSD test. P<0.05 was set as a statistically significant level. Different letters indicate significant difference.
