# Supplementary material for: Knock down of transforming growth factor beta improves expressions of co-stimulatory molecules, type I interferon-regulated genes, and pro-inflammatory cytokine in PRRSV-inoculated monocyte-derived macrophages
Source: BMC Vet Res. 2024 Aug 3;20:344. doi: 10.1186/s12917-023-03760-8 (PMC11297646; doi:10.1186/s12917-023-03760-8)
Supplement: Supplementary file 4 — Supplementary Material 4 [file 12917_2023_3760_MOESM4_ESM.docx]

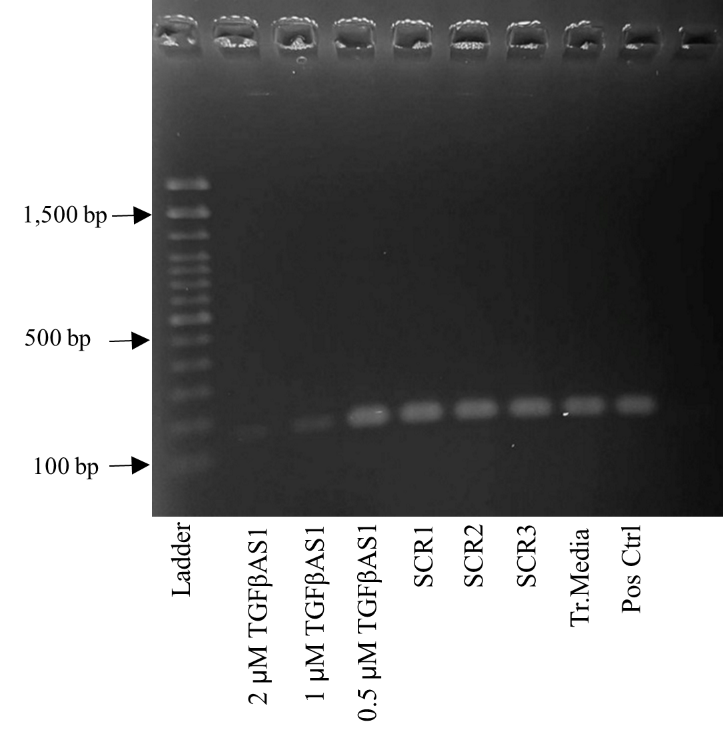


Additional file 2. Band intensities indicate the quality of TGFβ1 knockdown (refer to Figure 1E). Optimization of TGFβAS1 concentration for TGFβ1 mRNA knockdown. MDMs were transfected with TGFβAS1 (0.5, 1, or 2 µM) and stimulated with a mixture of poly I:C and LPS. MDMs transfected with Scr ODNs or treated with Tr. media alone prior to stimulation with a mixture of poly I:C and LPS served as Scr and Tr. media controls, respectively. Untransfected MDMs stimulated with a mixture of poly I:C and LPS served as Pos Ctrl.
