# Supplementary material for: Knock down of transforming growth factor beta improves expressions of co-stimulatory molecules, type I interferon-regulated genes, and pro-inflammatory cytokine in PRRSV-inoculated monocyte-derived macrophages
Source: BMC Vet Res. 2024 Aug 3;20:344. doi: 10.1186/s12917-023-03760-8 (PMC11297646; doi:10.1186/s12917-023-03760-8)
Supplement: Supplementary file 5 — Supplementary Material 5 [file 12917_2023_3760_MOESM5_ESM.docx]

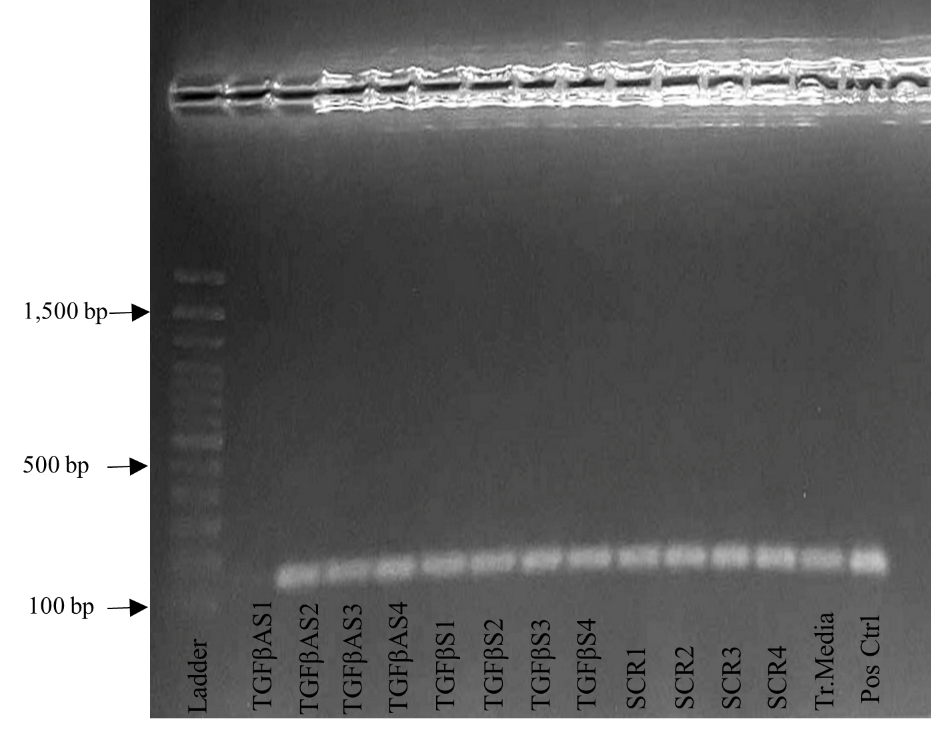


Additional File 1. Band intensities indicate the quality of TGFβ1 knockdown (refer to Figure 1D). Effect of TGFβ1 antisense (AS1-4), sense (S1-4) and scramble (Scr1-4) phosphorothioate-modified ODNs on expression of TGFβ1 mRNA in MDMs stimulated with a mixture of poly I:C and LPS. MDMs transfected with transfection media (Tr. media) alone and stimulated with a mixture of poly I:C and LPS served as Tr. media control. Untransfected MDMs stimulated with a mixture of poly I:C and LPS served as positive control (Pos Ctrl).
